# Prenatal stress modulates HPA axis homeostasis of offspring through dentate TERT independently of glucocorticoids receptor

**DOI:** 10.1038/s41380-022-01898-9

**Published:** 2022-12-08

**Authors:** Meng-Ying Liu, Lu-Lu Wei, Xian-Hui Zhu, Hua-Chen Ding, Xiang-Hu Liu, Huan Li, Yuan-Yuan Li, Zhou Han, Lian-Di Li, Zi-Wei Du, Ya-Ping Zhou, Jing Zhang, Fan Meng, Yu-Lin Tang, Xiao Liu, Chun Wang, Qi-Gang Zhou

**Affiliations:** 1 State Key Laboratory of Reproductive Medicine, Department of Clinical Pharmacology, School of Pharmacy, Nanjing Medical University, Nanjing, China; 2 Department of Pharmacy, The Affiliated Drum Tower Hospital of Nanjing University Medical School, Nanjing, China; 3 Department of Clinical Pharmacy, Sir Run Run Hospital, Nanjing Medical University, Nanjing, China; 4 Department of Psychiatry, Affiliated Nanjing Brain Hospital, Nanjing Medical University, Nanjing, China; 5 School of Applied Science, Temasek Polytechnic, Singapore, Singapore; 6 College of Pharmacy, Nanjing University of Chinese Medicine, Nanjing, China; 7 The Key Center of Gene Technology Drugs of Jiangsu Province, Nanjing Medical University, Nanjing, China

**Keywords:** Neuroscience, Psychology

## Abstract

In response to stressful events, the hypothalamic-pituitary-adrenal (HPA) axis is activated, and consequently glucocorticoids are released by the adrenal gland into the blood circulation. A large body of research has illustrated that excessive glucocorticoids in the hippocampus exerts negative feedback regulation of the HPA axis through glucocorticoid receptor (GR), which is critical for the homeostasis of the HPA axis. Maternal prenatal stress causes dysfunction of the HPA axis feedback mechanism in their offspring in adulthood. Here we report that telomerase reverse transcriptase (TERT) gene knockout causes hyperactivity of the HPA axis without hippocampal GR deficiency. We found that the level of TERT in the dentate gyrus (DG) of the hippocampus during the developmental stage determines the responses of the HPA axis to stressful events in adulthood through modulating the excitability of the dentate granular cells (DGCs) rather than the expression of GR. Our study also suggests that the prenatal high level of glucocorticoids exposure-induced hypomethylation at Chr13:73764526 in the first exon of mouse *Tert* gene accounted for TERT deficiency in the DG and HPA axis abnormality in the adult offspring. This study reveals a novel GR-independent mechanism underlying prenatal stress-associated HPA axis impairment, providing a new angle for understanding the mechanisms for maintaining HPA axis homeostasis.

## Introduction

Maternal exposure to stresses during pregnancy reprograms the hypothalamic–pituitary–adrenal (HPA) axis of the offspring [1, 2]. The HPA axis is a critical adaptive system of the body for facing with urgently physical and psychological challenges [3]. Although acute or short-term activation of the HPA axis is beneficial for reacting to stimuli, chronic or long-term activation causes harmful effects [4]. It has been well-documented over the last 40 years that hyperactivity of the HPA axis is highly associated with the pathology of adolescents’ depression [4]. Maternal stress-induced excessive glucocorticoids cross the placenta, impairing fetal HPA development and changing the HPA axis activity balance in the offspring [5–9]. However, the molecular mechanism underlying the gestational stress-induced fetal HPA axis dysfunction remains largely unknown.

The hippocampus is a major component exerting inhibitory modulation of the activity of the paraventricular nucleus (PVN) in the hypothalamus, playing an important role in maintaining the homeostasis of the HPA axis [10–12]. The endogenous receptor for glucocorticoids, glucocorticoid receptor (GR), is a widely expressed nuclear hormone receptor in the brain, with the highest density in the hippocampus [13, 14]. Activation of hippocampal GR exerts negative feedback modulation of the HPA axis activity by inhibiting the expression of corticotropin­releasing factor or hormone (CRF or CRH) in the PVN region, consequently causing decreased secretion of glucocorticoids from the adrenal cortex [11]. Decreased number and/or impaired function of the GR have been reported in patients with depression in clinic [15, 16]. Preclinical studies suggest that the dysfunction of GR in the PFC and hippocampus are involved in the chronic stress-induced attenuation of the negative feedback [17, 18]. The decreased expression and impaired function of GR are also implicated in the prenatal stress-caused elevated HPA axis reactivity in the offspring [7, 19, 20]. Accumulating evidences support the hypothesis that the impairment of the central stress hormone negative regulation system is causally involved in the development of depression [21]. However, a challenging question is whether there is a mechanism beyond GR for maintaining the homeostasis of the HPA axis in depression?

Telomerase are composed of the highly conserved catalytic telomerase reverse transcriptase (TERT), the telomerase RNA (TERC), and a suite of species-specific proteins [22]. Telomerase highly exists in the brain at embryonic stages and declines gradually after birth except in the adult stem cells [23]. Besides tumors and aging, telomerase is implicated in other central never system diseases [23]. More and more studies report a negative correlation between TERT activity or telomere length and stressful events [23, 24]. Accelerated telomere length attrition was observed in children experienced psychological stress and early adversity in the childhood [24]. Prenatal stress exposure in the intrauterine life caused subsequent shorter telomere length in young adulthood [25, 26]. In the present study we uncovered an important role of TERT in the dentate gyrus (DG) of the hippocampus in regulation of HPA axis activity without influencing GR expression. By designer receptors exclusively activated by designer drugs (DREADDs) transgenic mice, electrophysiology, and virus tools, we found that the level of dentate TERT during the brain developmental stage determines the response of the HPA axis to stressful events in adulthood through modulating the excitability of the dentate granular cells (DGCs) rather than regulating the GR expression. Mechanically, elevated glucocorticoids-induced DNA hypomethylation of CpG site (Chr13:73764526) in the exon 1 of *Tert* gene in the DG accounted for the repression of TERT level, accounting for the prenatal maternal stress-related HPA axis dysfunction in the adult offspring. The present study revealed a novel mechanism for maintaining the endogenous steady state of the HPA axis beyond the GR theory.

## Results

### *Tert* gene knockout causes HPA axis hyperactivity without GR deficiency

**Fig. 1:**
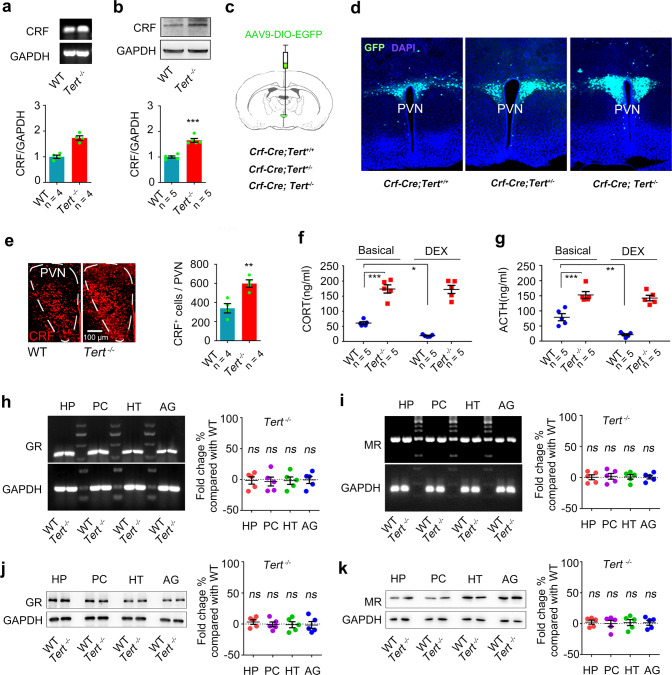
Hyperactivity of the HPA axis in *Tert* knockout mice without GR and MR deficiency. **a** RT-PCR showing the levels of CRF and GAPDH mRNA in the hypothalamus of *Tert*^*−/−*^ and WT mice. *n* = 4. Student’s *t* test. **b** Western blot showing the levels of CRF and GAPDH protein in the hypothalamus of *Tert*^*−/−*^ and WT mice. *n* = 5. Student’s *t* test. **c**, **d** Experimental design and representative images showing GFP^+^ cells in the PVN of *Crf-Cre; Tert*^*+/+*^, *Crf-Cre; Tert*^*+/*−^, or *Crf-Cre; Tert*^*−/−*^ mice. *n* = 3. **e** Immunofluorescence showing the number of CRF^+^ cells in the PVN of *Tert*^*−/−*^ and WT mice. *n* = 4. Student’s *t* test. **f** The concentrations of CORT in the plasma of *Tert*^*−/−*^ and WT mice treatment with DEX or vehicle. *n* = 5. Two-way ANOVA. **g** The concentrations of ACTH in the plasma of *Tert*^*−/−*^ and WT mice treatment with DEX or vehicle. *n* = 5. Two-way ANOVA. **h**, **i** RT-PCR showing the levels of GR, MR, and GAPDH mRNA in the HP, PC, HT, and AG of *Tert*^*−/−*^ and WT mice. *n* = 5. **j**, **k** Western blot showing the levels of GR, MR, and GAPDH protein in the HP, PC, HT, and AG of *Tert*^*−/−*^ and WT mice. *n* = 5. HP Hippocampus, PC Prefrontal cortex, HT Hypothalamus, AG Amygdala. *P < 0.05, **P < 0.01, and ***P < 0.001, *ns* indicates no significant difference. Error bars indicate s.e.m.

To know whether the TERT protein is implicated in the modulation of HPA axis activity, we measured the expression level of CRF, which is synthesized and secreted by neurons in the PVN region of the hypothalamus, in a strain of *Tert* gene knockout (*Tert*^−/−^) mice [27]. Western blot and reverse transcription polymerase chain (RT-PCR) measurements showed a higher level of CRF protein and mRNA content in the hypothalamus in the *Tert*^−/−^ mice than their wild-type littermates (WT) (Fig. 1a, b). To further measure the level changes of CRF in the PVN region after deletion of *Tert* gene, we generated transgenic mice (*Crf-Cre; Tert*^+/−^, *Crf-Cre; Tert*^−/−^, or *Crf-Cre; Tert*^*+/+*^ mice) by crossing the *Crf-Cre* and *Tert*^−/−^ mice. We injected AAV-DIO-GFP virus into the PVN region of these mice. The *Crf* promoter permits expression of a CRE recombination after deleting a stop codon flanked by 2 loxP sites, leading to expression of GFP reporter in CRF neurons in the PVN of these mice (Fig. 1c, sFig. 1a). A gene dose dependent response was supported by the number of GFP^+^ cells in the CRF region in *Crf-Cre; Tert*^−/−^, *Crf-Cre; Tert*^+/−^, and *Crf-Cre; Tert*^+/+^ mice (Fig. 1d, sFig. 1a). In line with this observation, immunofluorescence detection using anti-CRF, and anti-c-FOS primary antibodies revealed significantly increased numbers of CRF-positive cells and c-FOS-positive cells in the PVN region of *Tert*^−/−^ mice compared with WT mice (Fig. 1e, sFig. 1b). An elevated level of glucocorticoids in the plasma is a hallmark of enhanced activity of the HPA axis [18]. A significant higher level of corticosterone (CORT), the glucocorticoids in rodent, was examined by liquid chromatography-mass spectrometer (LC-MS) method in the plasma of *Tert*^−/−^ mice compared with WT mice (Fig. 1f, sFig. 1c). The dexamethasone (DEX) suppression test is a neuroendocrine test measuring the negative feedback effect of the HPA axis [18]. In the WT mice, the plasma level of CORT was reduced by DEX treatment; however, DEX administration failed to suppress the rise of CORT in the plasma of *Tert*^−/−^ mice (Fig. 1f). Consistently, the measurement of adrenocorticotropic hormone (ACTH) by Elisa assay showed a higher concentration of ACTH in the plasma of *Tert*^−/−^ mice, which was not suppressed by DEX treatment. While the level of ACTH in the plasma of WT mice was successfully downregulated after DEX treatment, indicating that ACTH secretagogue biosynthesis in the hypophysiotropic neurons was dysfunctional due to persist excessive CRF generation in the *Tert*^−/−^ mice (Fig. 1g). Collectively, these data demonstrated that TERT deficiency induced by *Tert* gene knockout causes hyperactivity of the HPA axis.

GR, in the hippocampus and hypothalamus, plays a key role in the negative feedback modulation of HPA axis activity, contributing to the HPA axis homeostasis [21]. Mineralocorticoid receptor (MR) in the limbic neurons also participates in the maintaining of the HPA axis homeostasis [21]. To know the paradigm of GR and MR expression in *Tert*^−/−^ mice, GR and MR mRNA and protein level were measured by RT-PCR and Western Blot. Surprisingly, *Tert* gene knockout did not influence the expression of GR and MR in the regions in relevant to depression including the hippocampus, prefrontal cortex, hypothalamus, and amygdala (Fig. 1h–k). These observations suggest that TERT is implicated in the regulation of the HPA axis in a GR- and MR- independent manner.

### TERT in the DG modulates response of the HPA axis to negative and positive stressors

**Fig. 2:**
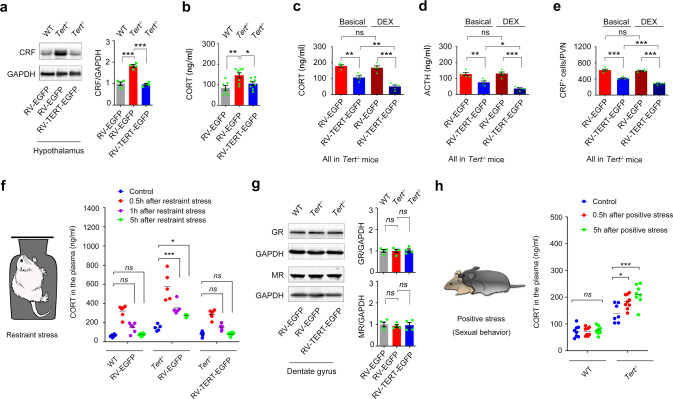
The level of dentate TERT determines the HPA axis activity without relevance to GR and MR. Western blot showing the levels of CRF and GAPDH in the hypothalamus (**a**) and the concentration of CORT in the plasma (**b**), of 5-week-old *Tert*^*−/−*^ and WT mice received microinjection of 1 μl of RV-TERT-EGFP or RV-EGFP into the bilateral DGs. Two months after virus injection, the samples for western blot were prepared and the concentration of CORT in the plasma was measured. *n* = 4–8. One-way ANOVA. The concentration of CORT (**c**) and ACTH (**d**) in the plasma, and the number of CRF cells (**e**) in the PVN of 5-week-old *Tert*^*−/−*^ mice received injection of 1 μl of RV-TERT-EGFP or RV-EGFP into the bilateral DGs. *n* = 5. One-way ANOVA. **f** In another cohort of 5-week-old *Tert*^*−/−*^ and WT mice, 1 μl of RV-TERT-EGFP or RV-EGFP was injected into the DGs. Two months after injection, mice were exposed to 1 h-restrain stress and the plasma were collected for CORT measurement at 0.5, 1, and 5 hours after the restrain stress exposure. *n* = 5–6. Two-way ANOVA. **g** Western blot showing the level of GR, MR, and GAPDH in the DG. The samples were prepared from mice in **a**. One-way ANOVA. **h** The concentration of CORT in the plasma of *Tert*^*−/−*^ and WT mice at different time points after sexual behavior. *P < 0.05, **P < 0.01, and ***P < 0.001, *ns* indicates no significant difference. Error bars indicate s.e.m.

In our previous research, we found that telomerase in the adult DG regulates depression-related behaviors [27, 28]. To evaluate the importance of dentate TERT deficiency in HPA activity dysfunction, we constructed a retrovirus encoding a full-length mouse *Tert* complementary DNA and an EGFP reporter gene linked by internal ribosome entry site DNA sequences (RV-TERT-EGFP) [29], and infused 1 μl of RV-TERT-EGFP or RV-EGFP (with a high titer at 1.0E + 8) into the DGs of the hippocampi of 5-week old *Tert*^−/−^ and WT mice which were at adolescent period (Fig. 2a, b). Retrovirus specifically infected dividing dentate neural stem cells (NSCs) those then generated neurons expressing EGFP and TERT two months later (sFig. 2a, b). Both measurement of CRF protein in the hypothalamus and examination of CORT level in the plasma demonstrated that replenishment of TERT in the developing DGs remarkably reversed the HPA axis dysfunction in *Tert*^−/−^ mice (Fig. 2a, b). Both DEX suppression test and ACTH measurement showed that DEX administration decreased the level of CORT in the plasma of *Tert*^−/−^ mice 2 months after re-expression of TERT in the DGs (Fig. 2c, d). The measurement of CRF^+^ cells in the PVN also demonstrated the same effect (Fig. 2e).

In adaption to acute stressful challenge, HPA axis is transiently activated, causing elevated glucocorticoids in the body. Increased glucocorticoids in the hippocampus then exert negative regulation of the HPA axis, maintaining the endogenous glucocorticoid rhythms [30]. To know whether TERT in the hippocampal DG implicates in this process, we infused RV-TERT-EGFP or RV-EGFP into the DGs of 5-week-old *Tert*^−/−^ and WT mice. Two months after injection, mice were exposed to 1 h-restrain stress and the plasma was collected at 0.5, 1, and 5 hours after the restrain stress exposure (Fig. 2f). The measurement of CORT in the plasma showed that the concentration of CORT increased at 0.5 h and 1 h and returned to normal at 5 h after stress in WT mice (Fig. 2f). Strikingly, the concentration of CORT elevated persistently at a higher level at all the time points and did not return to physiological level at 5 hours after stress in *Tert*^−/−^ mice. More importantly, re-expression of TERT in the DGs of *Tert*^−/−^ mice reversed all these changes (Fig. 2f). Additionally, GR and MR expression in the DGs was not affected (Fig. 2g). However, infection of RV-TERT-EGFP in the DGs of the hippocampi of 20-week-old *Tert*^−/−^ mice at adulthood failed to rescue the HPA axis impairment (sFig. 3). These results collectively indicated that TERT in the developing DG is critical for normal HPA axis function in the adulthood.

Sexual behavior is considered as a type of positive stressor or memory. To determine whether TERT affect the response of HPA axis to positive stressors, we measured the plasma levels of CORT in male WT and *Tert*^−/−−/−^ mice 0.5, 1, and 5 hours after sexual behavior (Fig. 2h). The plasma CORT remained unchanged in WT mice at different time points, indicating that HPA axis did not response to this type of positive stress under normal state. However, plasma CORT elevated at 0.5 and 5 hours after sexual behavior in *Tert*^−/−^ mice (Fig. 2h). Our data suggested that positive stimuli trigger intensive stressful reaction of the HPA axis in TERT-deficient state, contributing to HPA axis abnormality in the pathology of depression.

### TERT in the DG regulates HPA axis activity independent of GR

**Fig. 3:**
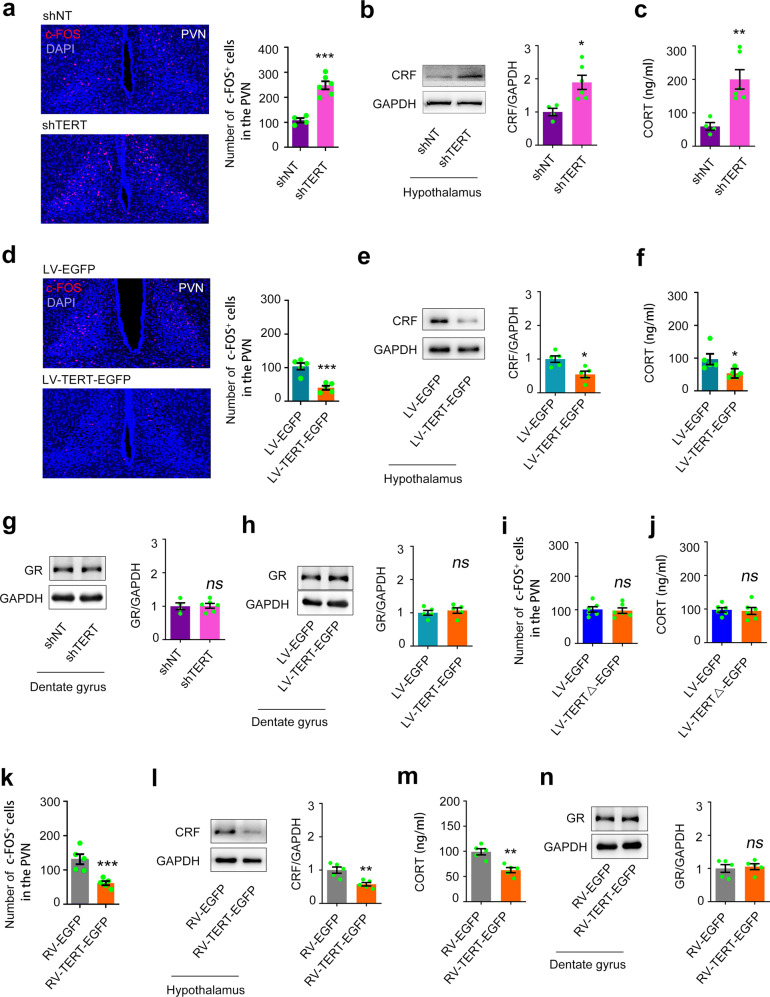
Telomerase catalytic activity is involved in the modulation of the HPA axis activity. The number of c-FOS^+^ cells in the PVN (**a**), the expression of CRF and GAPDH in the hypothalamus (**b**), and the concentration of CORT in the plasma (**c**), in C57BL/6 mice 2 months after injection of 1 μl of shTERT or shNT into the bilateral DGs. *n* = 4–6. Student’s *t* test. The number of c-FOS^+^ cells in the PVN (**d**), the expression of CRF and GAPDH in the hypothalamus (**e**), and the concentration of CORT in the plasma (**f**), in mice 2 months after injection of 1 μl of LV-TERT-EGFP or LV-EGFP into the bilateral DGs. *n* = 5. Student’s *t* test. The expression of GR in the DG 2 months after shTERT (**g**) or LV-TERT-EGFP infection (**h**). *n* = 4–6. Student’s *t* test. The number of c-FOS^+^ cells in the PVN (**i**) and the concentration of CORT in the plasma (**j**) in mice 2 months after injection of 1 μl of LV-TERTΔ-EGFP or LV-EGFP into the bilateral DGs. *n* = 6. Student’s *t* test. The number of c-FOS^+^ cells in the PVN (**k**), the expression of CRF and GAPDH in the hypothalamus (**l**), the concentration of CORT in the plasma (**m**), and the expression of GR in the DG (**n**), in mice 2 months after injection of 1 μl of RV-TERT-EGFP or RV-EGFP into the bilateral DGs. *n* = 5. Student’s *t* test. *P < 0.05, **P < 0.01, and ***P < 0.001, *ns* indicates no significant difference. Error bars indicate s.e.m.

To investigate the role of dentate TERT, we constructed LV-TERT-shRNA-GFP (shTERT) or non-targeting small hairpin RNA (shNT) [29] and infused into the DGs of 5-week-old C57BL/6j mice bilaterally and sacrificed these mice 2 months later (Fig. 3a–c, sFig. 2c). Selective knockdown of TERT in the DGs resulted in a significant increase in the number of c-FOS^+^ cells in the PVN region (Fig. 3a), excessive expression of CRF in the hypothalamus, and elevated CORT levels in the plasma (Fig. 3b, c). Oppositely, we constructed a recombinant lentiviral vector carrying the gene-encoding mouse TERT and EGFP reporter cDNA (LV-TERT-EGFP) [29] and infused into the DGs of 5-week-old C57BL/6j mice bilaterally and sacrificed these mice 2 months later (sFig. 2d, e). Overexpression of TERT in the DGs repressed the HPA axis activity, including a smaller number of c-FOS^+^ cells in the PVN region (Fig. 3d), reduced CRF synthesis in the hypothalamus (Fig. 3e), and elevated CORT levels in the plasma (Fig. 3f). Consistently, RV-TERT-EGFP infection in the DGs of 5-week-old C57BL/6j mice induced the similar effect (Fig. 3h–k). Most importantly, both TERT knockdown and overexpression did not change the expression of GR and MR in the DGs (Fig. 3g, h, sFig. 4a, b).

The function of TERT beyond its catalytic activity has been reported [31, 32]. To investigate whether the telomerase activity is required in regulating HPA axis activity, we constructed a recombinant lentivirus vector carrying the cDNA of TERT with mutation (LV-TERTΔ-EGFP), causing expression of non-activity TERT [29]. A volume of 1 μl of LV-TERTΔ-EGFP was injected into the DGs of the hippocampi of 5-week-old C57BL/6j mice those were sacrificed for measurement 2 months later. Expression of inactivated TERT in the DGs did not change neuronal activity of neurons in the PVN of the hypothalamus (Fig. 3i) and the concentration of CORT in the plasma (Fig. 3j). To further confirm the role of telomerase activity, Azidothymidine [3-azido-3-deoxythymidine (AZT)], an inhibitor of telomerase activity, or vehicle was pumped continuously into the developmental DGs of 5-week-old C57BL/6j mice using Alzet osmotic minipumps (0.5 mM, 0.25 μl/h) for 7 days (sFig. 5). The CRF expression in the hypothalamus and the CORT concentration in the plasma of mice was not altered 24 hours after administration (sFig. 5a, b) but significantly increased 2 months later (sFig. 5d, e). Consistently, western blot measurement demonstrated that GR expression was not changed 1 (sFig. 5c) or 56 (sFig. 5f) days after AZT exposure. The AZT experiments indicate that it requires long term to elicit effect on HPA axis after telomerase activity inhibition. Repeatedly, the MR expression in the DGs was not affected by telomerase activity inhibition (sFig. 4c).

Taken all, these data suggested that telomerase catalytic activity per se is not related to HPA axis dysfunction but the biological alterations in chronic phase after telomerase activity inhibition in the developing DG account for HPA axis abnormality. However, GR and MR are not involved in this process.

### Hypo-excitability of DGCs accounts for TERT-deficiency induced HPA axis impairment

**Fig. 4:**
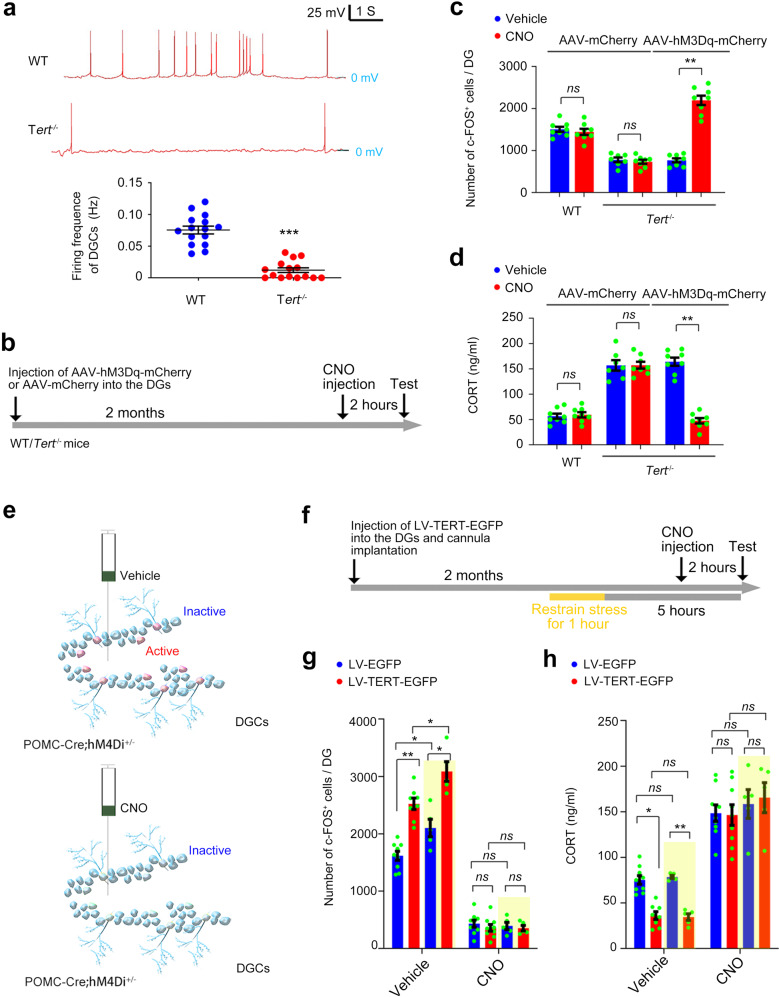
Hypo-excitability of DGCs following TERT deficiency impairs negative feedback regulation of the HPA axis. **a** Representative neuronal firing of DGCs of *Tert*^−/−^ and WT mice measured by whole-cell current clamp recording. The same results were observed in 15 individual DGCs from 3 mice in each group. Student’s *t* test. Experimental design (**b**) and analytical data showing the number of c-FOS^+^ cells in the DG (**c**) and the concentration of CORT in the plasma (**d**) of *Tert*^−/−^ and WT mice received microinjection of AAV-mCherry or AAV-hM3Dq-mCherry into the DGs. *n* = 7–8. Two-way ANOVA. Schematic experimental design and experimental paradigm for **g**–**h**. Analytical data showing the number of c-FOS^+^ cells in the DG (**g**) and the concentration of CORT in the plasma (**h**). *P < 0.05, **P < 0.01, ***P < 0.001, *ns* indicates no significant difference. Error bars indicate s.e.m.

Our previous study found that the development of DGCs is impaired in *Tert*^−/−^ mice. We then hypothesized that the excitability of the DGCs is repressed in *Tert*^−/−^ mice, and the consequence contributes to HPA axis abnormality. Indeed, whole-cell current clamp recording showed that neural firing rate of the DGCs was significantly reduced in *Tert*^−/−^ mice compared with WT mice (Fig. 4a). Engineered G protein–coupled human muscarinic acetylcholine M3 or M4 (hM3Dq or hM4Di) receptor expressed on the membrane of neurons do not react to any endogenous ligands. Upon the treatment of clozapine N-oxide (CNO), hM3Dq or hM4Di DREADDs receptors activate or suppress neuronal activity, respectively. To investigate the causal link between TERT and neuronal excitability of DGCs in regulation of HPA axis, we microinjected AAV-hM3Dq-mCherry or AAV-mCherry into the DGs of 5-week-old *Tert*^−/−^ and WT mice and administered clozapine-N-oxide (CNO) 1 month later (Fig. 4b). Upon administration of CNO (1 mg/kg, i.p.), DGCs were forcedly activated in the DG of *Tert*^−/−^ mice (Fig. 4c). In consistent, the HPA axis activity was repressed successfully upon administration of CNO in *Tert*^−/−^ mice received microinjection of AAV-hM3Dq-mCherry into the DGs (Fig. 4d).

Moreover, we crossed proopiomelanocortin (POMC)-Cre mice with ROSA26-hM4Di mice to generate POMC-Cre; hM4Di^f/+^ mice (sFig. 6a, b), in which POMC promoter drives Cre recombinase in developmental DGCs, leading to expression of hM4Di receptors in DGCs (sFig. 6b) [33]. Upon activation by CNO in the DGs of POMC-Cre; hM4Di^f/+^ mice, hM4Di receptors would suppress neuronal activity of DGCs (Fig. 4e) [34]. A volume of 1 μl of LV-TERT-EGFP or LV-EGFP was injected into the DGs of 5-week-old POMC-Cre; hM4Di^f/+^ mice. CNO or vehicle were infused into the bilateral DGs 2 months after virus infection (Fig. 4f). Overexpression of TERT significantly increased the number of c-FOS^+^ cells in the DG and decreased the CORT level in the plasma (Fig. 4g, h). Remarkably, the effects of TERT overexpression were blocked by CNO (10 μM, 1 μl) administration (Fig. 4g, h). Acute stressor stimulated the activity of DGCs and brought HPA axis action back to normal 5 hours later, which were facilitated by overexpression of TERT in the DG (Fig. 4g, h). Deactivation of DGs disrupted this negative feedback regulation system (Fig. 4g, h). These findings indicated that prenatal stress-induced deficiency of TERT and the consequent hypo-excitability of DGCs might contribute to abnormal HPA axis activity homeostasis in the offspring.

### Dentate TERT deficiency contributes to prenatal-stress induced HPA axis dysfunction of adult offspring

To investigate genetic association of functional polymorphisms in *Tert* gene and depression, we conducted a genetic association analysis of case-control samples in the Chinese population using single nucleotide polymorphism. The demographic and clinical features of the 259 patients with major depressive disorder (MDD) and 196 healthy controls (Hcs) were listed in Table 1. There were no statistically significant differences in age (*t* = 0.886, *P* = 0.378) and gender (χ^2^ = 3.8, *P* = 0.051). We did not find association between most nucleotide polymorphism site of h*Tert* and depression, excluding the rs2736099 site. The allele frequency in the two groups was in Hardy-Weinberg equilibrium. The distribution of the three genotype (AA, AG, GG) differed between the patients with MDD and the Hcs (*P* = 0.022, logistic regression adjusted for age and sex). As shown in Table 2, in patients with MDD, the mutation rate of A allele is significantly higher (*P* = 0.009). One-way ANOVA analysis revealed significant differences in the total score of Hamilton Depression Scale (*F* = 6.766, *P* = 0.001). The differences were also significant among HAMD factor 2 (weight, *F* = 3.049, *P* = 0.049), HAMD factor 3 (cognition, *F* = 4.14, *P* = 0.010), HAMD factor 5 (block, *F* = 3.16, *P* = 0.044), HAMD factor 6 (sleep symptoms, *F* = 6.534, *P* = 0.002) and HAMD factor 7 (hopelessness, *F* = 3.094, *P* = 0.040). The A-carriers of rs2736099 scored significantly in HAMD-1/2/3/5/6/7 and GG group. These results from clinical samples indicated that abnormal function of TERT relates with the pathology of depression.

**Table 1:** Demographic and clinical characteristics of total samples.

		MDD (*n* = *259*)	Hcs (*n* = *196*)	*t/u/c2*	*P*
Age (years)		40.78 ± 10.7	39.27 ± 8.5	0.886	0.378
Gender [*n* (%)]	Males	116 (105.9)	70 (80.1)	3.8	0.051
	Females	143 (153.1)	126 (115.9)		
Genotype [*n* (%)]	AA	49 (18.9%)	28 (14.3%)	7.674	**0.022**
	AG	130 (50.2%)	83 (42.3%)		
	GG	80 (30.9%)	85 (43.4%)		
	A	228 (44%)	139 (35.5%)	6.788	**0.009**
	G	290 (56%)	253 (64.5%)

**Table 2:** Association between h*Tert* rs2736099 and patients with MDD in HAMD scores.

	AA	AG	GG	F	P
Course	72.5 ± 164.8	66.9 ± 111.6	46.9 ± 51.7	1.076	0.343
Age	43.9 ± 11.9	41.7 ± 10.8	37.5 ± 8.9	6.63	**0.002**
HAMD	35.86 ± 10.9	35.7 ± 10.6	30.6 ± 9.7	6.766	**0.001**
Factorscore1 (anxiety)	9.51 ± 2.8	9.12 ± 3.0	8.63 ± 3.9	1.172	0.331
Factor score2 (weight)	1.02 ± 0.9	0.89 ± 0.8	0.66 ± 0.8	3.049	**0.049**
Factor score3 (cognition)	5.78 ± 3.4	5.98 ± 3.7	4.60 ± 3.1	4.14	**0.017**
Factor score4 (circadian rhythm)	0.73 ± 0.9	0.68 ± 0.9	0.5 ± 0.8	1.391	0.251
Factor score5 (block)	8.02 ± 2.3	8.08 ± 3.4	7.01 ± 2.0	3.16	**0.044**
Factor score6 (sleep symptom)	5.53 ± 2.8	5.35 ± 2.6	4.15 ± 2.4	6.534	**0.002**
Factor score7 (hopelessness)	5.27 ± 1.9	5.32 ± 2.3	4.58 ± 2.2	3.094	**0.047**

**Fig. 5:**
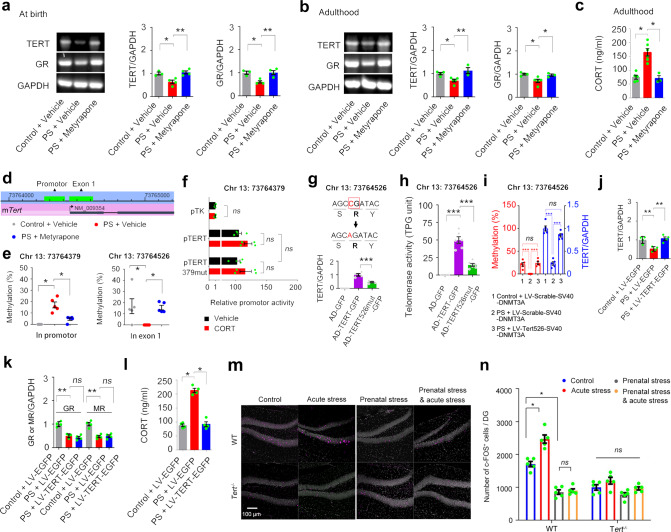
Prenatal stress-induced dentate TERT deficiency leads to dysfunctional HPA axis in adult offspring. **a** The mRNA levels of TERT, GR, and GAPDH in the hippocampus of newborn mice experienced prenatal stress. n = 3-4. One-way ANOVA. **b** The mRNA levels of TERT, GR, and GAPDH in the DG of 3-month-old offspring experienced prenatal stress. *n* = 4–5. One-way ANOVA. **c** The concentration of CORT in the plasma of 3-month-old offspring experienced prenatal stress. *n* = 3–6. One-way ANOVA. **d** CpG sites in the promotor and exons of mouse T*ert* gene. **e** Methylation levels of Chr13:73764379 and Chr13:73764526 in the DG of 3-month-old offspring experienced prenatal stress. *n* = 5. One-way ANOVA, Kruskal-Wallis test. **f** Luciferase experiments were performed in 293T cells transfected with control plasmid pTK, plasmid pTERT, or plasmid pTERT379mut. *n* = 4. Two-way ANOVA. Schematic design of the construction of AD-TERT526mut-GFP, and the TERT mRNA level (**g**) and activity (**h**) in the cultured hippocampal NSCs cells infected with AD-GFP, AD-TERT-GFP, or AD-TERT526mut-GFP. *n* = 4–6. One-way ANOVA. **i** The methylation level of Chr13:73764526 and the expression of TERT in the DG after selectively editing of DNA methylation of Chr13:73764379. A volume of 1 μl of LV-*Tert526*-SV40-DNMT3A or LV-*Scramble*-SV40-DNMT3A was microinjected into the DG of 5-week mice experienced prenatal stress. Two months later, the tests were performed. *n* = 5. One-way ANOVA. The mRNA level of TERT, GR, MR, and GAPDH in the DG (**j**, **k**) and the concentration of CORT in the plasma (**l**) of 3-month-old offspring infused LV-TERT-EGFP or LV-EGFP into the DG of 5 weeks old mice experienced prenatal stress. *n* = 4. One-way ANOVA. **m**, **n** Representative photos and analytical data showing the number of c-FOS^+^ cells in the DG of *Tert*^−/−^ and WT mice with or without prenatal stress. Acute stress (1-hour restraining) was performed 30 minutes before collection of the plasma and perfusion with 4% PFA. *n* = 5. Two-way ANOVA. *P < 0.05, **P < 0.01, ***P < 0.001, *ns* indicates no significant difference. Error bars indicate s.e.m.

Maternal stress and prenatal glucocorticoids exposure impair fetal HPA developmental programming [35–38]. To know the TERT expression level in the hippocampus of the offspring after prenatal stress, we measured TERT mRNA level in the hippocampus of newborn offspring and in the DG of 3-month-old offspring whose mother exposed to negative stressors including restraint and light stimuli for 14 days during pregnancy (Fig. 5a, sFig. 7). Prenatal maternal stress significantly reduced hippocampal mRNA level of dentate TERT in newborn offspring (Fig. 5a) and caused a persistently reduced level of TERT to adulthood (Fig. 5a). By administration of CORT synthesis inhibitor metyrapone (100 mg/kg, s.c., 1 time per day, 14 days) 30 minutes before stress exposure during pregnancy, we found that exposure to prenatal excessive glucocorticoids was the primary factor accounted for abnormal TERT expression in the DG (Fig. 5b) and HPA axis dysfunction (Fig. 5c) in the offspring.

DNA methylation of specific CpG sites in the promoter or exons of genes causes abnormal gene transcription in offspring experienced prenatal stress in the fetus [39]. There are 2 CpG islands containing 35 sites specifically in the promoter and exon 1 of *Tert* gene (Fig. 5d and sFig. 8). Methylation status of these sites in the DG of the adult offspring were measured via bisulfite amplicon sequencing (BSAS) analysis (sTable 1). Stress during prenatal period caused hypermethylation at Chr13:73764379 and hypomethylation at Chr13:73764526, which were prevented by inhibiting excessive glucocorticoids synthesis in the mother body (Fig. 5e, sTable 1). To understand the mechanism, we constructed the promoter of *Tert* gene (pTERT) or the promoter of *Tert* gene with site mutation at Chr13:73764379 (CG → AG) (pTERT379mut) into the luciferase assay. Luciferase experiments were performed in 293 T cells transected with control plasmid pTK, plasmid pTERT, or plasmid pTERT379mut. Unexpectedly, both CORT incubation and single nucleotide mutation at Chr13:73764379 did not influence the promoter activity of *Tert* gene (Fig. 5f), indicating non-involvement of this nucleotide in the modulation of the expression of TERT. Predictably, methylation level of this site is not relevant to TERT deficiency caused by maternal stress. Next, we studied the effect of hypomethylation at Chr13:73764526 on TERT transcriptional expression by constructing synonymous mutation (C → A) in adenovirus vector (AD-TERT526mut-GFP) (Fig. 5g). Then we cultured hippocampal NSCs cells from *Tert*^*−/−*^ mice and infected NSCs with AD-TERT-GFP, AD-TERT526mut-GFP, or AD-GFP for 4 days. None of TERT mRNA and activity was detected by RT-PCR and TRAP assay in AD-GFP-infected *Tert*^*−/−*^ NSCs. TERT expression and activity was significantly lower 4 days after infection with AD-TERT526mut-GFP in compared with AD-TERT-GFP infection (Fig. 5g, h), indicating that Chr13:73764526 hypomethylation is potentially implicated in abnormal expression of TERT. Furthermore, to selectively edit of DNA methylation of Chr13:73764526, we fused dCas9 with enzymes in the methylation/demethylation pathway by constructing LV-*Tert526*-SV40-DNMT3A or LV-*Scramble*-SV40-DNMT3A to target Chr 13:73764526 CpG without altering DNA sequence, based on TALE system [40, 41]. Two-month after microinjection into the DGs of 5-week-old mice experienced prenatal stress, the hypomethylation status of Chr 13:73764526 site of *Tert* and the TERT level in the DG was reversed (Fig. 5i). Moreover, microinjection of LV-TERT-EGFP normalized the level of TERT in the DG but not GR and MR (Fig. 5j–k), and as result, DG TERT replenishment counteracted the diverse effect of prenatal maternal stress on HPA axis function of the offspring (Fig. 5l).

In addition, the number of c-FOS^+^ cells in the granular layer of *Tert*^−/−^ mice is significantly lower in comparison to WT mice (Fig. 5m, n). Acute stress (1-hour restraining) enhanced the activity of DGCs in WT mice but not in *Tert*^−/−^ mice. Prenatal stress significantly suppressed the neuronal excitability of DGCs in WT mice but not *Tert*^−/−^ mice. Acute stressors in adulthood failed to activate DGCs in both WT and *Tert*^−/−^ mice experienced prenatal stress (Fig. 5m, n). These results suggested that dentate TERT deficiency contributes to prenatal stress-induced HPA axis dysfunction of adult offspring via affecting neuronal activity of DGCs.

## Discussion

A substantial body of clinical and preclinical evidence converges to suggest that conditions during the intrauterine period of life play an important role in developmental programming [35–38]. Fetal programming triggered by exposure to excessive glucocorticoids during prenatal stress impairs the development and subsequent lifelong function of the HPA axis of the offspring, causing persist adverse effects throughout the life [42]. GR has been illustrated by extensive literatures as an essential molecule in the modulation and functioning of the HPA axis [36, 37, 43]. In the present study, we found that *Tert* gene knockout caused hyperactivity of the HPA axis with normal GR and MR expression in the brain. Replenishment of TERT in the DG of *Tert*^*−/−*^ mice normalized the activity of HPA axis without GR and MR expression change. Overexpression of TERT protein in the developing DG repressed neuronal activation and CRF synthesis in the PVN of the hypothalamus while TERT knockdown in the developing DG enhanced neuronal activation and CRF synthesis in the PVN. However, telomerase catalytic activity per se is not the direct factor causing HPA axis dysfunction and the biological alteration in chronic phase induced by sustained low telomerase catalytic activity could account for the HPA axis impairment in the developing brain. Chemogenetic manipulation of the DGCs demonstrated that the level of TERT in the DG during the developmental stage determines the response of HPA axis to stressful event in adulthood via modulating the excitability of the DGCs rather than the expression of GR and MR. Most importantly, we revealed that prenatal maternal stress reduced TERT level, through hypomethylation at Chr13:73764526 in exon 1 of TERT gene, in the hippocampus of the newborn offspring and persisted in adulthood, contributing to HPA axis impairment independently of GR and MR.

It is revealed that epigenetic modifications of DNA by stress may contribute to the complex pathology of neuropsychiatric disorders [44]. Series of CpG sites with significantly different DNA methylation levels were detected in neonates exposed to maternal stress during pregnancy [39]. Specially, DNA hypermethylation or hypomethylation occurred in the DG contribute to stress-induced gene expression and depression-related behavior change [45, 46]. Genome-wide studies suggested that fetal glucocorticoids surge resulted in promoter-specific epigenetic changes in the fetal guinea pig hippocampus and most of the affected promoters exhibited hypomethylation [35, 47]. Here we found that the hypomethylation of the exon 1 in *Tert* gene correlated with lower level of TERT in the DG which caused dysfunctional HPA axis activity of mice experienced prenatal stress. In support of our finding, clinical prospective cohort studies showed that maternal stress during pregnancy negatively correlated with telomere length detected in umbilical cord blood [26, 48]. A post-mortem brain study demonstrated that a significant decrease in telomere length in the hippocampus of MDD subjects [49]. To our knowledge, no prior studies have investigated DNA methylation in *Tert* gene or linked it with HPA axis dysfunction. More importantly, the modulation of HPA axis by TERT is not related to GR which has been proved a key role in balancing HPA axis, implying a novel molecular mechanism for HPA axis homeostasis.

Acute stress resulted in activation of granule neurons in the DG demonstrated by detection of c-FOS and ARC immunoreactivity [50, 51]. In contrast, chronic stress decreased neuronal activity of granular cells in the DG [52]. Higher densities of c-FOS in the DG following acute stress also were observed in this study. By chemogenetic activating or silencing of DGCs, we found that neuronal activity of granular cells in the DG modulate the HPA axis activity. Interestingly, DGCs derived from *Tert*^*−/−*^ NSCs displayed hypo-excitability documented by c-FOS immunoreactivity and electrophysiological examination. Prenatal stress-induced deficiency of TERT in NSCs could be a causal factor for nonreactive state of DGCs to acute stress stimulation. Our previous studies found that TERT loss induced impaired neurogenesis, neural development including dendrite development and neuritogenesis, and neural circuit integration [28, 29]. Accordingly, the reduced input connection could be the reason of low neuronal activity of DGCs after downregulation of TERT in the DG. Prenatal stress caused atrophy of the hippocampus and damage of neurogenesis in the DG of juvenile rhesus monkeys [53]. Prenatal stress altered dendrite morphology and synaptic connectivity in the prefrontal cortex and hippocampus of developing offspring [54]. TERT deficiency could also contribute to maternal-stress-induced neurodevelopment abnormality associated with reduced learning, memory, attention, and intelligence during childhood.

Nowadays, the latest concept of ‘stress’ has been refined as conditions where an environmental demand exceeds the natural regulatory capacity of an organism [55]. Koolhaas and his/her colleagues emphasize that the physiological response and neuroendocrine reaction to ‘real stress’ should be unpredictable and uncontrollable [55]. Although previous study showed that sexual behavior increased CORT level in rodents [55–57], our data demonstrated that the HPA axis did not react to sexual behavior, a type of rewarding/appetitive stimuli. Surprisingly, rewarding stimuli were transformed into averse conditions to activate the HPA axis in *Tert*^*−/−*^ mice. Neuroendocrine reaction of the HPA axis to rewarding stimuli could be an important pathology of depression and dentate TERT functions in this process. Telomerase is present at high levels in the developing brain [56]. Our evidence suggests that fetal reprogramming of TERT during prenatal stress leads to impaired homeostatic processes of the HPA axis, including hyper-reaction to aversive stress and transformation of rewarding stimuli into aversive stress.

The fetal HPA axis is developed during pregnancy but is still reshaped during early life [58]. Our data showed that re-expression of TERT, in the DG of young mice experienced prenatal stress, normalized the HPA axis function independently of glucocorticoid receptors including GR and MR. This study proposes a novel molecular mechanism connecting fetal HPA axis programming and subsequent health outcomes.

## Methods

### Mice

In this study, neonatal, 5-week-old, or 8-week-old *Tert*^−/−^ mice and the littermates, produced by intercrossing *Tert*^+/−^ mice, are used for experiments. The *Tert*^+/−^ mice were backcrossed to C57BL/6J mice over more than ten generations. *Pomc*-Cre (Tg(Pomc1-cre))16Lowl/J (Stock No: 005965), *Crf*-Cre (Stock No: 012704), and ROSA26-hM4Di (Stock No: 026219) mice were purchased from The Jackson Laboratory. All mice were housed 5 per cage under standard laboratory conditions (22 ± 1 °C, 60% humidity, 12-h light/dark cycle with lights on at 07:00, food and water ad libitum) for one week before starting experiments. All the experiments were approved by the Institutional Animal Care and Use Committee of Nanjing Medical University (protocol number: IACUC-1704010).

### Drug

Clozapine N-oxide was purchased from Sigma-Aldrich and DMSO (0.5%) in saline was used as a vehicle. Azidothymidine [3-azido-3-deoxythymidine (AZT)] and Metyrapone were purchased from Sigma-Aldrich. Dexamethasone was purchased from China National Pharmaceutical Group Corporation.

### Virus and plasmid

RV-TERT-EGFP and RV-EGFP The sequence of TERT-EGFP in the plasmid pDC315-TERT-EGFP was cut by using restriction enzymes and inserted into the plasimd pCAG-EGFP (For RV-EGFP) by T4 DNA ligase to replace the sequence of EGFP in pCAG-EGFP. The plasmid was named as pCAG-TERT-EGFP, which was used to identify by DNA sequencing. The primer sequences were as follows: forward, 5′-GAGGATCCCCGGGTACCGGTCGCCACCATGACCCGCGCTCCTCGTTGCC-3′; reverse, 5′-TCCTTGTAGTCCATACCGTCCAAAATGGTCTGAAAGTCTGTGCTTAG-3′. The PCR fragments and the pGV287-GFP plasmid were digested with Age 1 and BamH 1 and ligated with T4 DNA ligase to produce RV-TERT∆-GFP. Using 100 μl Lipofectamine 2000, 293 T cells were co-transfected with 22.5 μg of pCAG-TERT-EGFP, 15 μg of pCMV-GP, and 7.5 μg of pCMV-VSVG to generate the recombinant retrovirus, RV-TERT-EGFP. After 48 h, supernatant was harvested from 293 T cells, filtered at 0.45 μ m, and pelleted by ultracentrifugation at 18000 × *g* for 2 h at 4 °C. After resuspension by PBS, serially diluted retrovirus was used to transduce 293T cells; 4 days later, labeled 293T cells were counted to calculate the viral titer (~2 × 107 transducing units/ml). As a control, we also generated a retroviral vector that expresses GFP (RV-EGFP) alone [29].

LV-TERT-shRNA-GFP Lentiviral particles of LV-TERT-shRNA-GFP and its control shRNA were purchased from Santa Cruz, CA, USA. LV-TERT-shRNA-GFP is a pool of concentrated, transduction-ready viral pa rticles containing 4 target-specific constructs that encode 19- 25 nt (plus hairpin) shRNA designed to knock down gene expression. Each vial contains 200 μl frozen stock containing 1.0 × 106 infectious units of virus (IFU) in Dulbecco’s Modified Eagle’s Medium with 25 mM HEPES pH 7.3 [29].

LV-TERT-EGFP and LV-EGFP Briefly, the coding sequence of mouse TERT was amplified by RT-PCR. The primer sequences were as follows: forward, 5′-GTAGAACGCAGATCGAAT-TCATGACCCGCGCTCCTCG-3′; reverse, 5′-CCCTTGCTCACCATG-AATTCGTCCAAAATGGTCTGAAAGTC-3′. The PCR fragments was cutted by restriction enzymes and inserted into the plasmid pCMV-GFP (For LV-EGFP) by T4 DNA ligase to replace the sequence of EGFP in pCMV-EGFP. EGFP was under control of another promotor pUbi. Mice mTERT△ gene was amplified by our previous constructed plasmid LV-TERT-EGFP carrying the whole gene encoding TERT by deletion mutant PCR. The primer sequences were as follows: forward, 5′-GAGGATCCCCGGGTACCGGTCGCCACCATGACCCGCGCTCCTCGTTGCC-3′; reverse, 5′-TCCTTGTAGTCCATACCGTCCAAAATGGTCTGAAAGTCTGTGCTTAG -3′. The PCR fragments and the pGV287-GFP plasmid were digested with Age 1 and BamH 1 and ligated with T4 DNA ligase to produce LV-TERT△-EGFP. Using 100 μl Lipofectamine 2000, 293 T cells were co-transfected with 20 μg of pCMV-TERT-GFP, 10 μg of VSVG, 7.5 μg of RSV-REV and 3.5 μg pMDL g/p RRE to generate the recombinant lentiovirus, LV-TERT-EGFP. After 48 h, supernatant was harvested from 293 T cells, filtered at 0.45 μ m, and pelleted by ultracentrifugation at 18,000 × *g* for 2 h at 4 °C. After resuspension by PBS, serially diluted retrovirus was used to transduce 293 T cells; 4 days later, labeled 293 T cells were counted to calculate the viral titer (~2 × 109 transducing units/ml). As a control, we also generated a retroviral vector that expresses GFP alone (LV-EGFP).

AD-TERT-GFP, AD-TERT526mut-GFP, and AD-GFP Briefly, the coding sequence of mouse TERT was amplified by RT-PCR. The primer sequences were as follows: forward, 5′-GTAGAACGCAGATCGAAT-TCATGACCCGCGCTCCTCG-3′; reverse, 5′-CCCTTGCTCACCATG-AATTCGTCCAAAATGGTCTGAAAGTC-3′. The PCR fragments and the pDC315-GFP plasmid were digested with EcoR I and ligated with T4 DNA ligase to produce pDC315-TERT-IRES-GFP [29]. The plasmid was used to trans-form competent DH5α Escherichia coli bacterial strains for identification. Using 100 μl of Lipofectamine 2000 mixed with 50 μl of DMEM, HEK293 cells were co-transfected with 5 μg of the pDC315-GFP plasmid with a cDNA encoding mTERT and 5 μg of the pBHG lox ∆E1,3 Cre plasmid as a helper plasmid to generate the recombinant adenovirus, AD-TERT-GFP. After 8 d, supernatant was harvested from HEK293 cells. After 3 times the virus amplification, the supernatant was filtered at 0.45 μm and purified using the Adeno-X Virus Purification kit. After resuspension, serially diluted adenovirus was used to transduce HEK293 cells. Seven days later, labeled HEK293 cells were counted to calculate the viral titer (~2.5 × 10 10 pfu/ml). As a control, we also generated a adenoviral vector that expresses GFP alone (AD-GFP). By mutant PCR, the nucleotide at Chr13:73764379 was replaced (CG → AG) (pTERT379mut), as pAD-TERT526mut-GFP. The plasmid was used to trans-form competent DH5α Escherichia coli bacterial strains for identification [29].

LV-*Tert526*-SV40-DNMT3A or LV-*Scramble*-SV40-DNMT3A A sequence-specific guide RNA (gRNA) (GCTGCGCAGCCGATACCGGG) was designed and constructed in lentivirus to target dCas9-Dnmt3a to Chr 13:73764526 site of mouse *Tert* gene, mediating modification of DNA methylation status without altering the DNA sequence. LV-*Scramble*-SV40-DNMT3A was used as control.

### Western blot analysis

Western bolt analysis of samples from cultured hippocampal neurons and hippocampal tissues of animals was performed as described previously [18]. In this study, we dissected the DG from the hippocampus for Western blot. The primary antibodies were as follows: mouse anti-GAPDH (1:4000; Santa Cruz Biotechnology), rabbit anti-corticotrophin-releasing factor (anti-CRF; 1:500; Santa Cruz Biotechnology). Rabbit anti-GR (1:200; Santa Cruz Biotechnology), Rabbit anti-MR (1:500; Santa Cruz Biotechnology). Appropriate horseradish peroxidase-linked secondary antibodies were used for detection by enhanced chemiluminescence (Thermo Fisher Scientific; USA). When we prepared the samples, all the hippocampus was checked to find the needle track of infusion. Only the samples with obvious needle track of infusion needles were remained for further measurement.

### RNA extraction and reverse transcription PCR

Total RNA was extracted from the DG of the hippocampus using Trizol reagent according to the manufacture’s instructions (Sigma-Aldrich; USA). The primers for TERT: forward, 5′ –ATGGCGTTCCTGAGTATG-3′, reverse, 5′-AGCCAGAGGCCTTTAGT-3′; For GR: forward, 5′-AGCAGAGAATGACTCTAC-3′, reverse, 5′-GAATTCAATACTCATGGAC-3′; For GAPDH: Forward, 5′-CAAGGTCATCCATGACAACTTTG-3′ and Reverse 5′-GTCCACCACCCTGTTGCTGTAG-3′. PCR conditions were 30 cycles of denaturation at 94 °C for 45 s, annealing at 55 °C for 45 s, and extension at 72 °C for 45 s. PCR products were separated by electrophoresis through 1.5% agarose gel containing 0.5% μg/mL ethidium bromide and imaged using a BioDoc-IT imaging system (Bio-Rad, USA); band intensities were determined using GS-710 calibrated imaging Densitometer (Bio-Rad, USA). The mRNA for GAPDH was included in the PCR mixture as a standard.

### Prenatal stress

Two stressors, restraint and light stimuli were applied to female pregnant mice for 3 × 45 min per day during pregnant day 7-21. For restraint, mice were maintained in a transparent Plexiglas cylinder (5 cm inner diameter) under a bright light (6500 lux). The container maintained the mice in a standing position without compression of the body. Each pregnant mother was single-housed and randomly assigned to control or stress groups. Control mothers were left undisturbed. After weaning at postnatal day 21, male offspring were housed and used for later experiments. Only 1 pup or 1-2 adult offspring per litter was used in both control and prenatal stress group.

### Positive stress

Male WT and *Tert*^*−/−*^ mice and female C57BL/6J mice without sexual experience were used for this study. Each C57BL/6J female mouse was placed in a mating cage (40 × 60 × 40 cm) of polycarbonate for 30 minutes. Then a male WT or *Tert*^*−/−*^ mouse was put into a mating cage and the sexual behavior was monitored by an experimenter. The male mice were sacrificed by another experimenter for measurement of CORT in the plasma at 0.5 or 5 h after a sexual behavior of mice. The experiences were performed during 9:00–11:00 AM.

### Stereotaxic injection

The surgical procedure was performed as described previously [29], with additional changes to the injection coordinates. Mice were anesthetized using isoflurane (3% induction, 1.5% maintenance) and fixed in a motorized stereotaxic apparatus at a rate of 0.2 µl /min (Stoelting, 53311). Stereotactic surgery was performed to deliver virus or solution into the dentate gyrus using the following coordinates: AP = − 2.42 mm; ML = 2.57 mm; DV = 3.32 mm. For the osmotic pump infusion, a 7d Alzet osmotic minipump containing AZT (6.7 μg/50 μl for 7d) was placed subcutaneously in the back of the animals, and a brain infusion cannula connected to the pump was positioned at the following coordinates: 2.42 mm posterior to bregma, 2.57 mm lateral to the midline, and 3.32 mm below dura. The infusion rate was 0.25 μl/h. Body temperature was maintained throughout the procedure using a heating pad and erythromycin eye ointment was applied to the eyes to prevent corneal drying. Mice with bleeding after withdrawal of the needle were excluded from the experiments.

### Electrophysiology

Brain slices were prepared with a Vibratome (VT1200s, Leica) in ice-cold, oxygenated artificial CSF (aCSF) containing 110 mM NaCl, 0.5 mM CaCl2, 2.5 mM KCl, 7 mM MgCl2, 1.3 mM NaH2PO4, 1.3 mM Na-ascorbate, 0.6 m M Na-pyruvate, 25 mM NaHCO3, and 20 mM Glucose, and then incubated in warm oxygenated aCSF (34 °C) for 1 hour. Brain slices (350 μm) containing the hippocampal region were transferred into the recording chamber and super-fused (2 ml/min) with oxygenated aCSF at room temperature (22–25 °C). Whole-cell patch recordings were performed with a computer-controlled amplifier (MultiClamp 700B, Molecular Devices). The pipettes for cell-attached recording (3–4 MΩ) contained bath solution and the seal resistance was ∽200 MΩ. GFP-positive neurons were targeted in the DG area under the visual guidance of green, fluorescent signals using an upright microscope (Examiner Z1, Zeiss). CNO (10 μM) dissolved in aCSF was released to the recording area with a small pressure using an 8-channel drug-delivery system (MPS-1, Inbio Life Science Instrument).

### Luciferase assays

Firefly and Renilla luciferase activities were quantified in lysates using the Dual Luciferase Reporter Assay kit (Promega) on a Victor3 1420 multilabel counter (PerkinElmer) according to the manufacturer’s recommendations. Luciferase readings were corrected for background, and Firefly luciferase values were normalized to Renilla to control for transfection efficiency.

### Cell Cultures

NSCs were cultured from embryonic hippocampus, on embryonic day 18 (E18), of pregnant female *Tert-/-* mice, mated with male *Tert-/-* mice, in neurobasal medium (Gibco) containing 20 ng/ml bFGF, 20 ng/ml EGF, and 2% B27 supplement as reported. All cultures were maintained in an incubator (HERAcell 150, Thermo Fisher Scientific, Waltham, MA, http://www.thermofisher.com) with humidified atmosphere of 95% air and 5% CO2 at 37°C.

### CORT and ACTH measurement

For CORT and ACTH level measurement, mice were decapitated between 9:00 and 12:00 A.M. Blood from angulus oculi vessels was collected in heparinized tubes. The CORT level was measured by a simple, highly sensitive and specific method based on ultra-fast liquid chromatography–tandem mass spectrometry method which has been developed and applied by our lab for the quantitation of corticosterone in mouse plasma. The lower quantification limit for corticosterone was 1 ng/mL [59]. ACTH in plasma was measured with a corticosterone ELISA kit according to the instructions of the manufacturer (PHOENIX PHARMACEUTICALS, INC).

### Telomerase activity assay

Telomerase activity was detected using TRA-PEZE XL telomerase detection kit (Millipore, Billerica, MA) as described previously [28]. Following the manufacturer’s instructions, telomeric repeat amplification protocol reactions (TRAP) were performed using the TRAPEZE XL telomerase detection kits (Millipore) for analysis of the telomerase activity. The fluorescence energy transfer primers were used to generate fluorescently labeled TRAP products, quantitatively measured with a fluorescence plate reader (SpectraMax M2e) or visualized after terminal deoxynucleotidyl transferase-mediated dUTP nickend labeling on a 10% nondenaturing gel and SYBR Green I (Invitrogen) staining.

### Immunohistochemistry

Serial hippocampal or hypothalamus sections (40 μm) were made on an oscillating tissue slicer. For c-FOS and CRF immunofluorescence, the sections were incubated with rabbit anti-c-FOS antibody (1:400; Synaptic Systems) and anti-CRF (1:200; Santa Cruz Biotechnology) in 0.1 M PBS with 3% goat serum and 0.3% Triton X-100, and binding was visualized with a Cy3-conjugated secondary antibody (1:200; Jackson immunoresearch). Images of immunostained neurons in all groups were captured with a Zeiss Axio Cam MRC 5(D) camera mounted on Carl Zeiss Axio Observer A1 microscope under same conditions. An experimenter coded all slides from the experiments before quantitative analysis. All CRF-positive cells and c-FOS-positive in the PVN were counted in each section by another experimenter blinded to the study code.

### Participants

Patients (*n* = 259) were recruited from the Nanjing brain hospital affiliated with Nanjing Medical University through in-patient department. All subjects met the DSM-V diagnosis of depression. The selection criteria for subjects were as follows: (1) 20–50 years old. (2) The scores of patients on the Hamilton Depression Rating Scales should 7 ≤score <24. (3) No other illnesses, no psychotherapy or medical treatment in the past 6 months or at most one treatment which the treatment time is not more than half a year. The study was approved by the Ethics Committee of the Affiliated Nanjing Brain Hospital, Nanjing Medical University. All subjects obtained informed consent.

### DNA extraction and genotyping

The 3 SNPs of hTERT were selected including rs33954691, rs2736099, rs2736118 with minor allele frequencies in the Chinese Han population greater than 0.3 selected from the 1000 Genomes database (https://www.ncbi.nlm.nih.gov/variation/tools/1000genomes/). All polymorphisms genotyped were in Hard-Weinberg equilibrium (HWE, *P* > 0.05). DNA was extracted from venous blood of all subjects. After the amplification of polymorphism-spanning fragments by multiplex PCR, samples were genotyped for the NOS1AP SNPs using a MassARRAY Sequenom assay. All primers were searched for dbSNP FASTA file in NCBI and designed using Agena software.

### Statistic

All data were analyzed using GraphPad Prism 8 (GraphPad Software). All experimental results are shown as mean ± s.e.m. After a homogeneity test of variance, when equal variances were assumed, unpaired or paired two tailed *t* tests or multiple two-tailed *t* tests with FDR correction were performed for comparison between two groups, one-way ANOVA was performed for comparison among three or four groups and two-way ANOVA was used to evaluate possible differences between groups with Bonferroni’s multiple comparisons test. The significance level for all tests was set at *P* < 0.05 or *Q* < 0.05. This study complies with randomization. The investigator was blinded to the group allocation during the experiment and/or when assessing the outcome.

## Supplementary information

Supplementary Information

sFigure 1

sFigure 2

sFigure 3

sFigure 4

sFigure 5

sFigure 6

sFigure 7

sFigure 8

sTable 1

Raw Data
