# Supplementary material for: Prenatal stress modulates HPA axis homeostasis of offspring through dentate TERT independently of glucocorticoids receptor
Source: Mol Psychiatry. 2022 Dec 8;28(3):1383–95. doi: 10.1038/s41380-022-01898-9 (PMC10005958; doi:10.1038/s41380-022-01898-9)
Supplement: Supplementary file 1 — Supplementary Information [file 41380_2022_1898_MOESM1_ESM.docx]

**Supplementary information**

Number of supplementary figures: 8

Number of supplementary tables: 1

**Supplementary figure legend**

**Supplementary figure 1**| **TERT knockout caused increased number of c-FOS^+^ in the PVN**

**a.** Representative images showing GFP^+^ cells in the PVN of *Crf-Cre; Tert^+/+^*, *Crf-Cre; Tert^+/-^*, or *Crf-Cre; Tert^-/-^* mice. AAV-DIO-GFP virus were injected into the PVN region of these mice 1 month before observation. The amplified version of the box regions were shown in the Figure 1d. **b.** Representative immunofluorescence photos and data showing the number of c-FOS^+^ cells in the PVN of *Tert^-/-^* and WT mice. n=5. Student's *t* test. **c**. Representative LC-MS measurement showing elevated CORT in the plasma of *Tert^-/-^* mice compared with WT mice. ****P* < 0.001. Error bars indicate s.e.m.

**Supplementary figure 2**| **DG infected with virus expressing TERT.**

**a.** Representative photo showing a slice of DG infected RV-TERT-EGFP 2 month after virus injection. **b.** Data graph showing the level of TERT in the DG 1 month after microinjection of RV-EGFP or RV-TERT-EGFP in *Tert^-/-^* and WT mice, measured by RT-qPCR. n=5. One-way ANOVA. **c.** Data graph showing the level of TERT in the DG 1 month after microinjection of shNT or shTERT in WT mice, measured by RT-qPCR. n=5. Student's *t* test. **d.** Representative photo showing a slice of DG infected LV-TERT-EGFP 2 months after virus injection. **e.** Data graph showing the level of TERT in the DG 1 month after microinjection of LV-EGFP or LV-TERT-EGFP in WT mice, measured by RT-qPCR. n=5. Student's *t* test. ***P* < 0.01, ****P* < 0.001. Error bars indicate s.e.m.

**Supplementary figure 3**| **Replenishment of TERT in the DG of *Tert^-/-^* mice at adulthood fails to normalize HPA axis.**

**a-b**. Western blot analysis data showing the level of CRF and GAPDH protein in the hypothalamus (**a**), and LC-MS measurement data showing the concentration of CORT in the plasma (**b**), of 20-week-old *Tert^-/-^* and WT mice received injection of 1 μl of RV-TERT-EGFP or RV-EGFP into the bilateral DGs. Two months after virus injection, the samples for western blot were prepared and the concentration of CORT in the plasma were measured. n=4. One-way ANOVA. ***P* < 0.01, ****P* < 0.001, *ns* indicates no significant difference, One-way ANOVA. Error bars indicate s.e.m.

**Supplementary figure 4**| **MR in the DG is not implicated in the HPA axis regulation by TERT.**

**a**. Western blot data showing the level of MR and GAPDH protein in the DG 1 month after microinjection of LV-EGFP or LV-TERT-EGFP into the DG of WT mice. n=5. Student's *t* test. **b**. Western blot data showing the level of MR and GAPDH protein in the DG 1 month after microinjection of LV-EGFP or LV-TERT-EGFP into the DG of WT mice. n=5. Student's *t* test. **c**. Western blot data showing the level of MR and GAPDH protein in the DG 1 month after infusion of AZT or vehicle using Alzet osmotic minipumps (0.5 mM, 0.25μl/h) for 7 days into the DG of WT mice. n=4-5. Student's *t* test. *ns* indicates no significant difference. Error bars indicate s.e.m.

**Supplementary figure 5**| **Telomerase activity inhibition in developmental DG impair HPA axis in a delayed manner.**

**a-c**. Western blot data showing the level of CRF and GAPDH protein in the hypothalamus (**a**), as well as the level of GR and GAPDH protein in the DG (**c**), and the concentration of CORT in the plasma (**b**) 24 hours after AZT exposure by Alzet osmotic minipumps (0.5 mM, 0.25μl/h) in the DG of 5-week-old mice for 7 days. n=10. Student's *t* test. **d-f**. Western blot data showing the level of CRF and GAPDH protein in the hypothalamus (**d**), as well as the level of GR and GAPDH protein in the DG (**f**), and the concentration of CORT in the plasma (**e**) 2 months after AZT exposure by Alzet osmotic minipumps (0.5 mM, 0.25μl/h) in the DG of 5-week-old mice for 7 days. n=4-5. Student's *t* test. **P* < 0.05, ***P* < 0.01, ****P* < 0.001, *ns* indicates no significant difference. Error bars indicate s.e.m.

**Supplementary figure 6**| **Validation of *POMC-Cre* mice and *POMC-Cre; hM4Di; YFP^fl/+^* mice.**

**a**. Representative photo showing the immunofluorescence of Cre in the DG of *POMC-Cre* mice. **b**. Representative photo showing the distribution of YFP^+^ cells in the brain of *POMC-Cre; hM4Di; YFP^fl/+^* mice. Note that DGCs were selectively expressed YFP.

**Supplementary figure 7**| **Prenatal stress** **schedule and design for grouping.**

**a**. Schematic schedule for prenatal stress experiments. Mothers were exposed to mild stressors including restraint and light stimuli during the last two weeks of pregnancy with or without Metyrapone (100 mg/kg, s.c., 1 time per day, 14 days) administration 30 minutes before stress exposure. **b**. Schematic design for grouping offspring with or without prenatal stress. One newborn pup was used per litter for Figure 5a, and 1-2 male 5-week old or adult offspring were used for Figure 5b,c, i, j, k, l, m, n.

**Supplementary figure 8**| **The CpG sites within mouse *Tert* gene.**

**a**. The distribution of CpG island in the promoter and exons of mouse *Tert* gene. **b**. The CpG sites in the promoter and exons of mouse *Tert* gene. Red color indicated that Chr13:73764379 locate in the promoter and Chr13:73764526 locate in the exon 1 of mouse *Tert* gene. The information was analyzed from PubMed database.
