# Supplementary figures and images for: Prenatal stress modulates HPA axis homeostasis of offspring through dentate TERT independently of glucocorticoids receptor
Source: Mol Psychiatry. 2022 Dec 8;28(3):1383–95. doi: 10.1038/s41380-022-01898-9 (PMC10005958; doi:10.1038/s41380-022-01898-9)

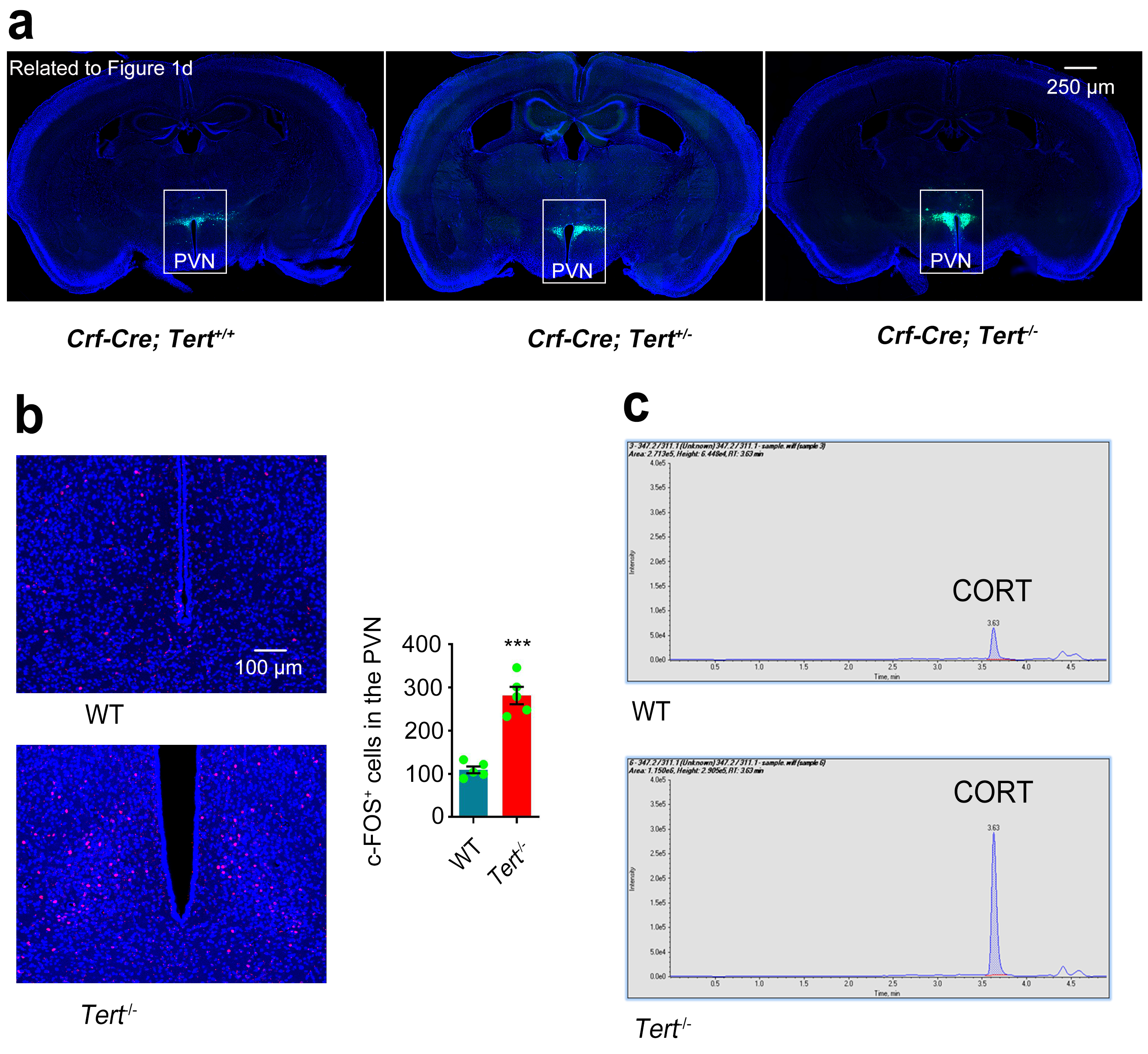

Supplement: Supplementary file 2 — sFigure 1 [file 41380_2022_1898_MOESM2_ESM.tif]

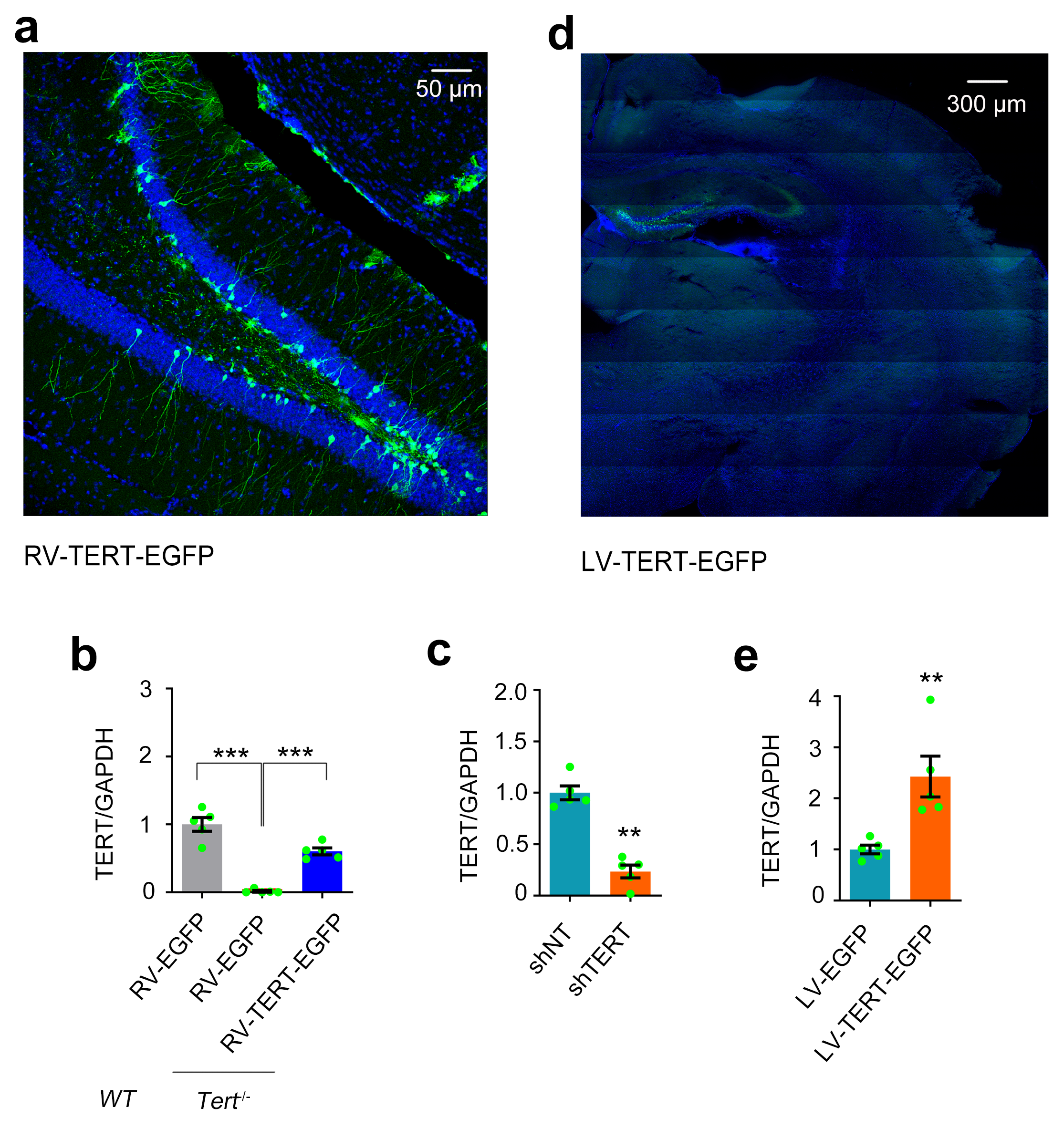

Supplement: Supplementary file 3 — sFigure 2 [file 41380_2022_1898_MOESM3_ESM.tif]

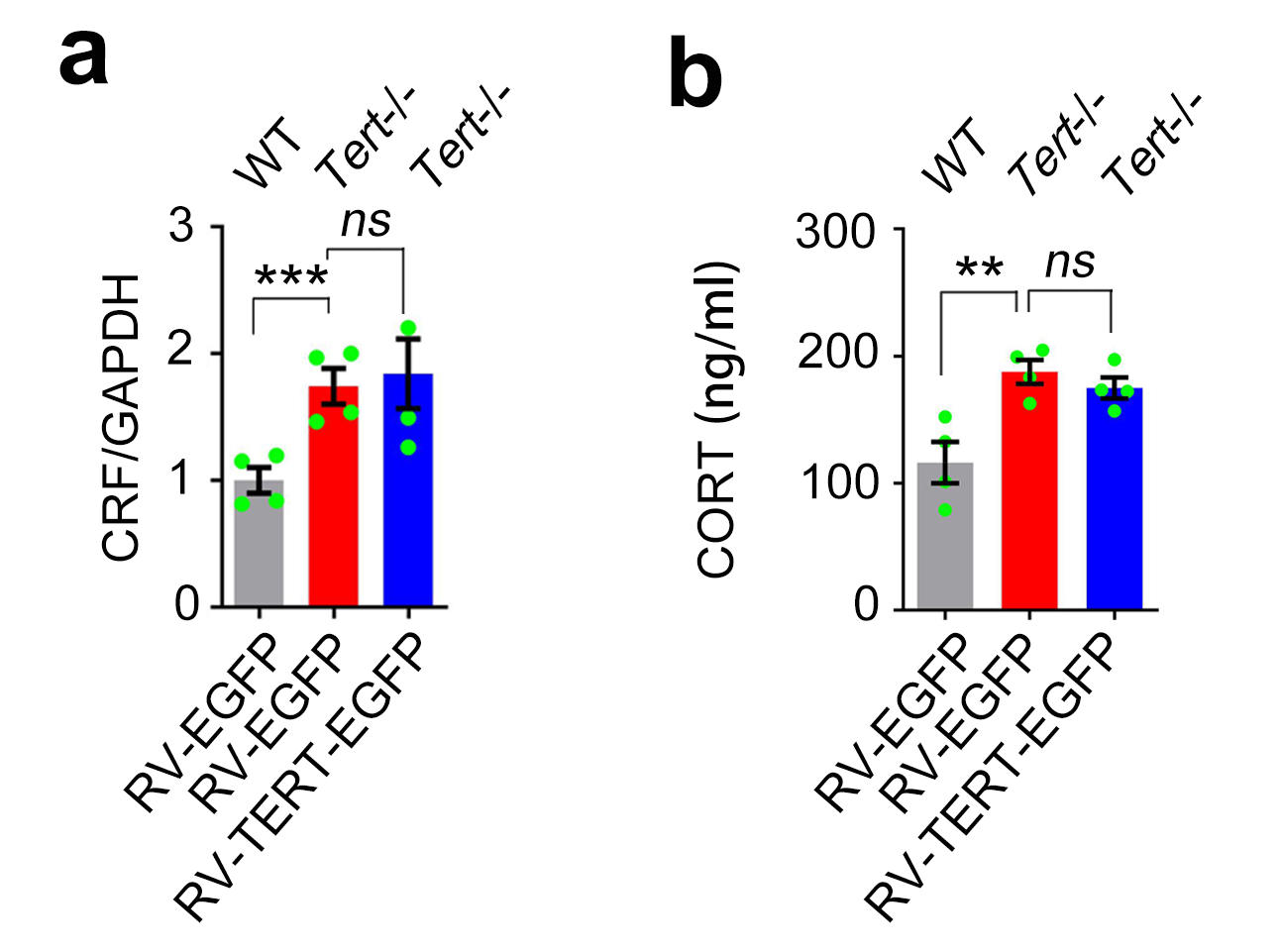

Supplement: Supplementary file 4 — sFigure 3 [file 41380_2022_1898_MOESM4_ESM.tif]

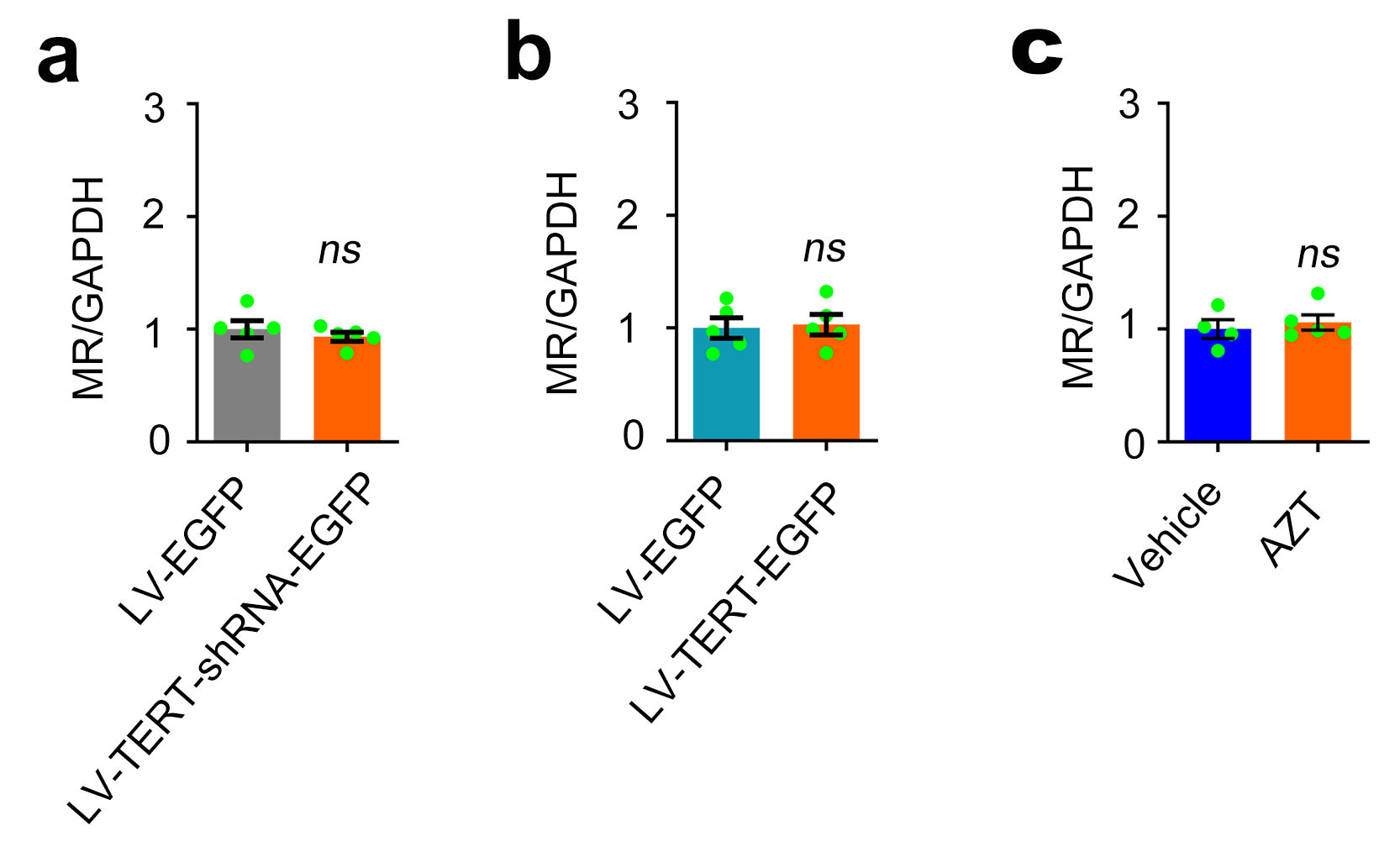

Supplement: Supplementary file 5 — sFigure 4 [file 41380_2022_1898_MOESM5_ESM.tif]

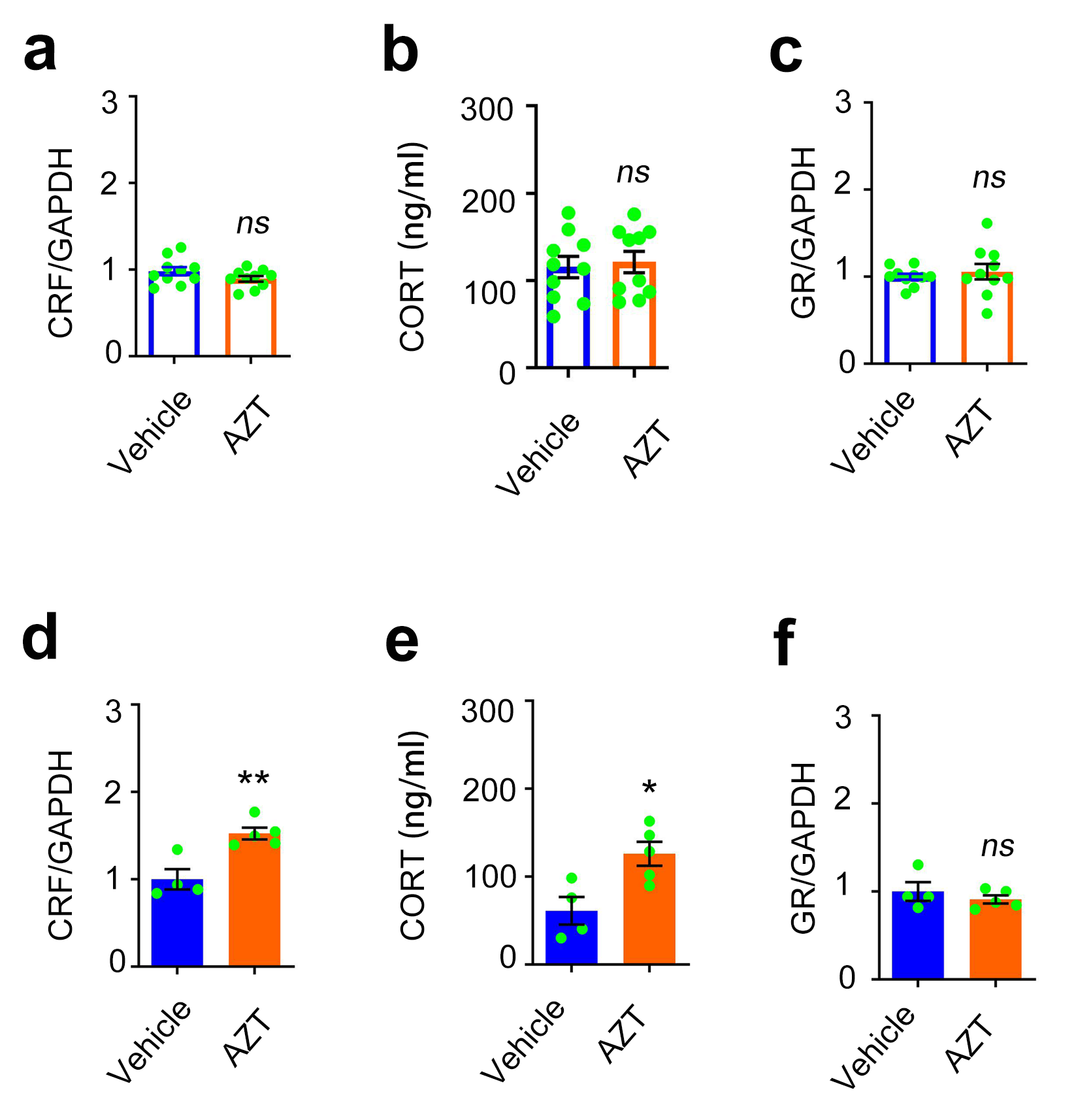

Supplement: Supplementary file 6 — sFigure 5 [file 41380_2022_1898_MOESM6_ESM.tif]

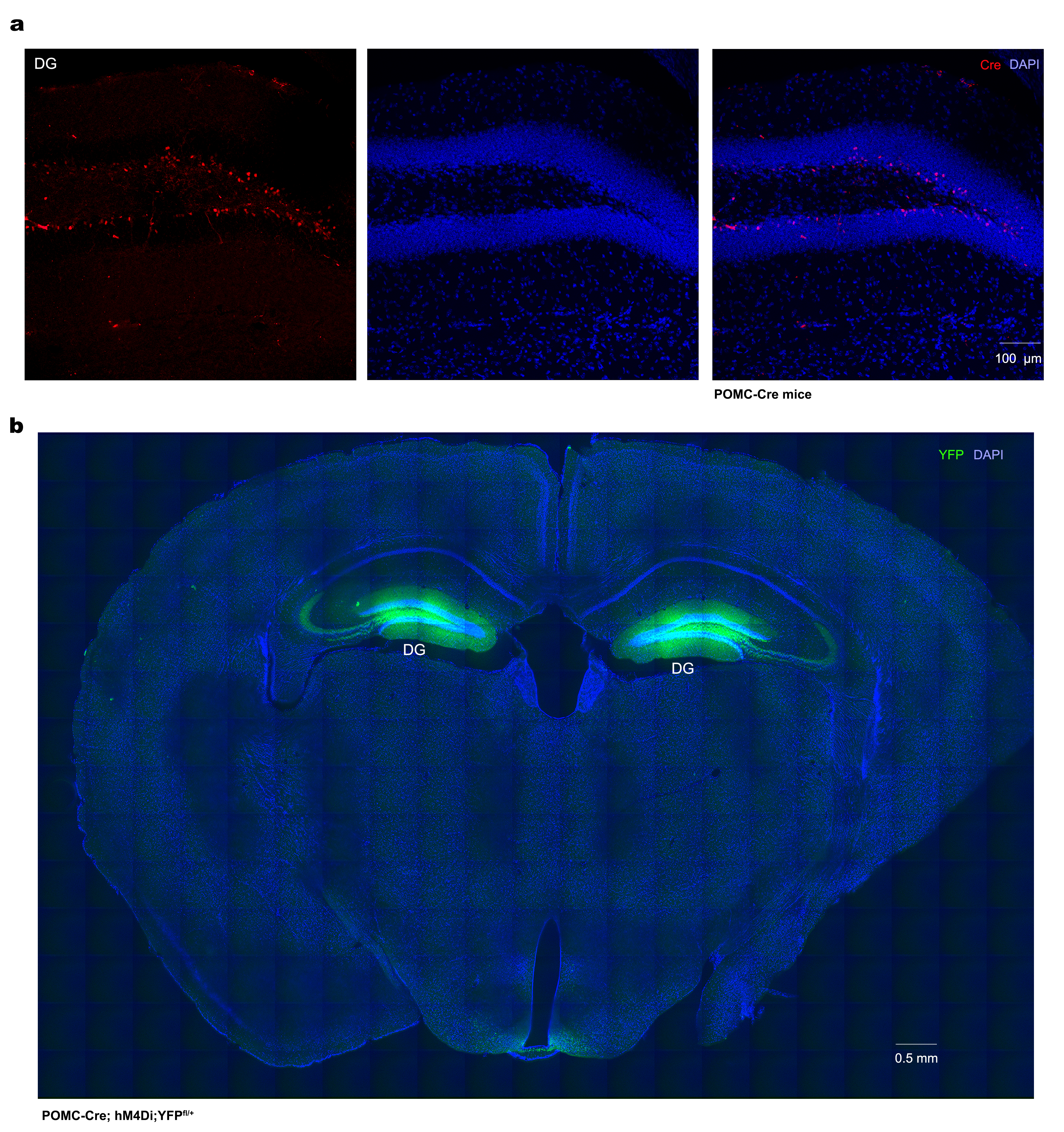

Supplement: Supplementary file 7 — sFigure 6 [file 41380_2022_1898_MOESM7_ESM.tif]

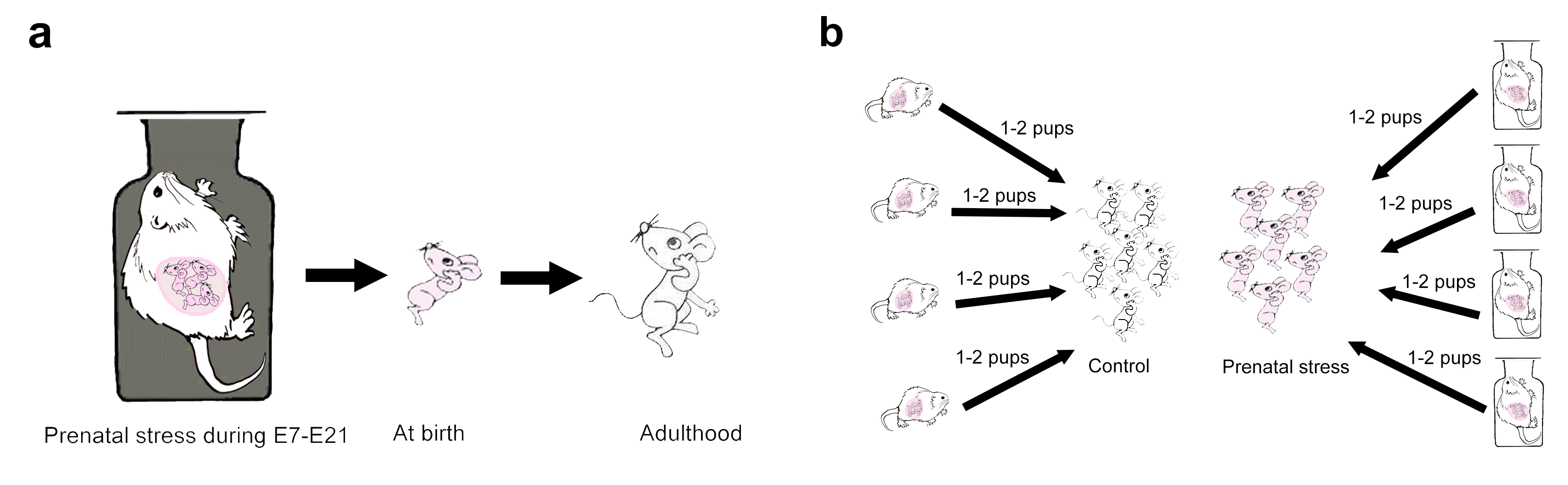

Supplement: Supplementary file 8 — sFigure 7 [file 41380_2022_1898_MOESM8_ESM.tif]

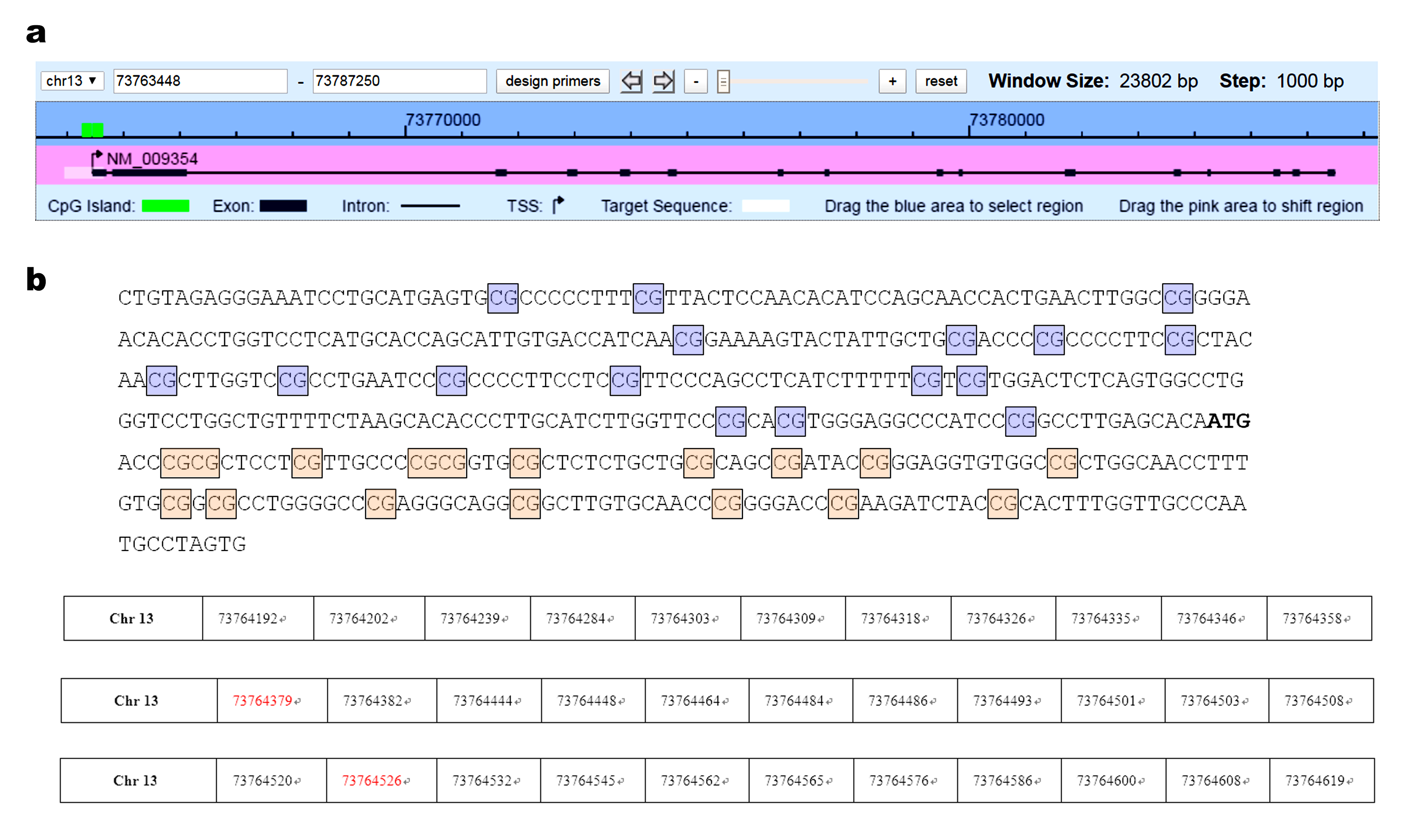

Supplement: Supplementary file 9 — sFigure 8 [file 41380_2022_1898_MOESM9_ESM.tif]
