# Supplementary material for: Prenatal stress modulates HPA axis homeostasis of offspring through dentate TERT independently of glucocorticoids receptor
Source: Mol Psychiatry. 2022 Dec 8;28(3):1383–95. doi: 10.1038/s41380-022-01898-9 (PMC10005958; doi:10.1038/s41380-022-01898-9)
Supplement: Supplementary file 10 — sTable 1 [file 41380_2022_1898_MOESM10_ESM.docx]

**Supplementary table 1**| **Methylation of mouse *Tert* gene measured by BSAS**

| **CpG site** | **Con-1** | **Con-2** | **Con-3** | **Con-4** | **Con-5** | **PS-1** | **PS-2** | **PS-3** | **PS-4** | **PS-5** | **PM-1** | **PM-2** | **PM-3** | **PM-4** | **PM-5** |
| --- | --- | --- | --- | --- | --- | --- | --- | --- | --- | --- | --- | --- | --- | --- | --- |
| 73764192 | 5.2 | 1.9 | 15.5 | 13.2 | 15.6 | 1.9 | 6.2 | 2.4 | 5.9 | 4.7 | 6.5 | 15.4 | 15.2 | 40.8 | 6.5 |
| 73764202 | 7.9 | 28.2 | 38.7 | 15.7 | 20.5 | 23.4 | 22.3 | 7.4 | 21 | 7.2 | 8.8 | 10.6 | 18 | 6.5 | 6.7 |
| 73764239 | 1.4 | 9.9 | 6 | 5 | 2.5 | 0 | 0 | 15.4 | 6.1 | 0 | 13.6 | 12.5 | 12.6 | 31.5 | 4.6 |
| 73764284 | 7.2 | 2 | 0 | 7.8 | 2.6 | 6.3 | 10 | 18.2 | 3.2 | 0 | 0 | 0 | 2.5 | 2.9 | 1.2 |
| 73764303 | 8.5 | 6.1 | 1.6 | 3.9 | 3.9 | 0 | 0 | 8.3 | 6.3 | 0 | 12 | 15 | 1.3 | 9.2 | 4.8 |
| 73764309 | 18.9 | 15.2 | 53.3 | 17.2 | 40.4 | 26.5 | 16.1 | 24.8 | 12.2 | 32.2 | 6.8 | 7.2 | 17.2 | 16.2 | 4.6 |
| 73764318 | 13.6 | 19.8 | 49.6 | 13.9 | 6.3 | 4.2 | 33 | 5.2 | 15.7 | 7.6 | 10.6 | 16.7 | 17.8 | 101.3 | 24.1 |
| 73764326 | 1.5 | 4.2 | 13 | 1.4 | 2.8 | 0 | 3.6 | 2.9 | 0 | 19.2 | 2.5 | 4.1 | 1.4 | 5.4 | 1.3 |
| 73764335 | 8.9 | 2.2 | 4.9 | 4.2 | 2.7 | 13.4 | 0 | 2.8 | 3.4 | 2.7 | 3.7 | 6.1 | 5.5 | 4.4 | 7.8 |
| 73764346 | 5.5 | 12.1 | 3 | 3.9 | 3.8 | 4.2 | 3.4 | 5.2 | 0 | 13 | 3.6 | 0 | 3.8 | 4.1 | 4.8 |
| 73764358 | 1.4 | 0 | 3 | 1.3 | 3.8 | 0 | 3.5 | 2.6 | 0 | 2.6 | 1.2 | 1.9 | 1.3 | 2.9 | 1.2 |
| 73764379 | 0 | 0 | 0 | 0 | 0 | 28 | 20 | 42 | 31 | 50 | 11 | 0 | 12 | 10 | 12 |
| 73764382 | 2.8 | 0 | 0 | 0 | 1.3 | 0 | 0 | 0 | 0 | 0 | 1.2 | 1.9 | 1.3 | 1 | 0 |
| 73764444 | 0 | 2.1 | 0 | 2.7 | 1.3 | 2.2 | 0 | 0 | 0 | 5.3 | 2.4 | 3.9 | 3.9 | 1 | 0 |
| 73764448 | 1.5 | 0 | 1.7 | 0 | 2.8 | 0 | 0 | 2.9 | 0 | 0 | 1.3 | 4.1 | 1.4 | 2.2 | 0 |
| 73764464 | 0 | 0 | 0 | 3 | 0 | 0 | 0 | 0 | 0 | 0 | 0 | 0 | 1.4 | 0 | 0 |
| 73764484 | 1.4 | 0 | 1.5 | 1.3 | 0 | 2.1 | 0 | 0 | 0 | 0 | 2.4 | 0 | 2.6 | 1 | 2.4 |
| 73764486 | 1.4 | 2 | 0 | 0 | 0 | 2.1 | 0 | 0 | 0 | 0 | 1.2 | 0 | 0 | 0 | 0 |
| 73764493 | 1.4 | 1.9 | 1.5 | 2.6 | 1.3 | 0 | 3.4 | 2.6 | 0 | 0 | 2.4 | 0 | 0 | 15.2 | 1.2 |
| 73764501 | 0 | 0 | 6.3 | 0 | 0 | 0 | 0 | 0 | 3.3 | 2.6 | 1.2 | 1.9 | 1.3 | 12.4 | 0 |
| 73764503 | 0 | 0 | 0 | 0 | 0 | 0 | 3.5 | 0 | 0 | 0 | 0 | 0 | 0 | 2 | 0 |
| 73764508 | 4 | 0 | 3 | 3.8 | 1.2 | 0 | 0 | 5 | 0 | 0 | 2.3 | 1.8 | 3.7 | 0 | 1.2 |
| 73764520 | 2.7 | 9.7 | 3 | 2.5 | 4.9 | 0 | 3.3 | 10.2 | 10.6 | 0 | 0 | 0 | 1.2 | 4.9 | 3.6 |
| 73764526 | 14 | 41 | 16 | 0 | 13 | 0 | 0 | 0 | 0 | 0 | 12 | 20 | 13 | 21 | 12 |
| 73764532 | 4.1 | 2 | 0 | 0 | 0 | 0 | 3.3 | 0 | 0 | 5.3 | 3.6 | 0 | 0 | 1 | 3.7 |
| 73764545 | 1.3 | 26.7 | 8.7 | 2.5 | 9.6 | 5.9 | 6.5 | 5 | 3 | 4.8 | 7.9 | 7.1 | 13.4 | 0.9 | 1.1 |
| 73764562 | 4 | 1.9 | 7.4 | 8.7 | 4.8 | 6.1 | 0 | 17.4 | 12.2 | 2.4 | 5.8 | 3.6 | 13.6 | 32.3 | 3.4 |
| 73764565 | 2.6 | 5.6 | 4.2 | 2.4 | 7 | 0 | 6.2 | 0 | 0 | 3.3 | 3.3 | 3.5 | 4.8 | 1.9 | 1.1 |
| 73764576 | 1.3 | 11.2 | 0 | 6.1 | 5.9 | 2 | 0 | 5 | 6 | 4.8 | 1.1 | 1.8 | 4.8 | 13.1 | 4.5 |
| 73764586 | 6.5 | 3.7 | 14 | 9.7 | 7 | 0 | 12.5 | 19.3 | 6 | 0 | 5.5 | 0 | 7.1 | 18.4 | 4.4 |
| 73764600 | 2.5 | 0 | 1.4 | 1.2 | 0 | 9.7 | 0 | 4.8 | 2.9 | 2.4 | 1.1 | 0 | 3.5 | 0.9 | 5.5 |
| 73764608 | 1.3 | 3.7 | 14.2 | 3.6 | 5.8 | 0 | 6.2 | 9.7 | 3 | 0 | 2.2 | 7 | 10.7 | 1.8 | 3.4 |
| 73764619 | 8.9 | 27.1 | 14.1 | 6 | 39 | 27 | 9.4 | 41 | 2.9 | 11.8 | 17.6 | 15.6 | 5.8 | 10.1 | 26.4 |

Methylation level of CpG site within the promoter and exons of mouse *Tert* gene in DGCs in the DG of the adult offspring exposed to prenatal stress. Con: Control+Vehicle; PS: Prenatal stress+Vehicle; PM: Prenatal stress+ Metyrapone.
